# Investigating host-microbiome interactions by droplet based microfluidics

**DOI:** 10.1186/s40168-020-00911-z

**Published:** 2020-10-01

**Authors:** Alexandra S. Tauzin, Mariana Rangel Pereira, Liisa D. Van Vliet, Pierre-Yves Colin, Elisabeth Laville, Jeremy Esque, Sandrine Laguerre, Bernard Henrissat, Nicolas Terrapon, Vincent Lombard, Marion Leclerc, Joël Doré, Florian Hollfelder, Gabrielle Potocki-Veronese

**Affiliations:** 1 TBI, CNRS, INRAE, INSAT, Université de Toulouse, F-31400 Toulouse, France; 2 Department of Biochemistry, University of Cambridge, Cambridge, CB2 1GA UK; 3 CAPES Foundation, Ministry of Education of Brazil, BrasÍlia, DF 70040-020 Brazil; 4 Drop-Tech, Canterbury Court, Cambridge, CB4 3QU UK; 5 CNRS, UMR 7257, Aix-Marseille Université, F-13288 Marseille, France; 6 USC 1408 AFMB, INRAE, F-13288 Marseille, France; 7 Department of Biological Sciences, King Abdulaziz University, Jeddah, Saudi Arabia; 8 Micalis Institute, INRAE, AgroParisTech, Université Paris-Saclay, F-78350 Jouy-en-Josas, France; 9 Metagenopolis, INRAE, F-78350 Jouy-en-Josas, France

**Keywords:** Functional metagenomics, Droplet microfluidics, Human gut microbiota, Human glycans, Beta-N-acetyl-galactosaminidase

## Abstract

**Background:**

Despite the importance of the mucosal interface between microbiota and the host in gut homeostasis, little is known about the mechanisms of bacterial gut colonization, involving foraging for glycans produced by epithelial cells. The slow pace of progress toward understanding the underlying molecular mechanisms is largely due to the lack of efficient discovery tools, especially those targeting the uncultured fraction of the microbiota.

**Results:**

Here, we introduce an ultra-high-throughput metagenomic approach based on droplet microfluidics, to screen fosmid libraries. Thousands of bacterial genomes can be covered in 1 h of work, with less than ten micrograms of substrate. Applied to the screening of the mucosal microbiota for β-N-acetylgalactosaminidase activity, this approach allowed the identification of pathways involved in the degradation of human gangliosides and milk oligosaccharides, the structural homologs of intestinal mucin glycans. These pathways, whose prevalence is associated with inflammatory bowel diseases, could be the result of horizontal gene transfers with *Bacteroides* species. Such pathways represent novel targets to study the microbiota-host interactions in the context of inflammatory bowel diseases, in which the integrity of the mucosal barrier is impaired.

**Conclusion:**

By compartmentalizing experiments inside microfluidic droplets, this method speeds up and miniaturizes by several orders of magnitude the screening process compared to conventional approaches, to capture entire metabolic pathways from metagenomic libraries. The method is compatible with all types of (meta)genomic libraries, and employs a commercially available flow cytometer instead of a custom-made sorting system to detect intracellular or extracellular enzyme activities. This versatile and generic workflow will accelerate experimental exploration campaigns in functional metagenomics and holobiomics studies, to further decipher host-microbiota relationships.

Video Abstract

## Background

The intestinal ecosystem plays a major role in keeping us healthy. Gut microbes interact with the host at the mucosal barrier, which physically protects the intestinal epithelium from direct contact with commensals, but also constitutes a nutrient-rich micro-habitat. However, excessive degradation of mucus, especially of the glycans that decorate mucins, has a significant impact on susceptibility to pathogens. Alteration of the mucus layer is also associated with inflammatory bowel diseases (IBD) including Crohn’s disease and ulcerative colitis [1–5]. Experimental characterization of host-microbiota interactions will help to understand the molecular mechanisms leading to IBD better.

In order to metabolize the structurally diverse glycans found in the intestine (host glycans, dietary glycans and, to a minor extent, microbial exopolysaccharides), bacteria produce a large panel of carbohydrate-active enzymes (CAZymes), which can be secreted, cell-surface associated, periplasmic or cytoplasmic [6, 7], and a battery of proteins to specifically sense, bind and transport glycans into cells for their complete breakdown. These metabolic machineries are often encoded by multigenic clusters [8], termed polysaccharide utilization loci (PULs) in *Bacteroidetes* [9]. Several plant glycan utilization pathways from gut bacteria have been characterized recently at the molecular level ([10–14] reviewed in [13]). However, biochemical studies can only be carried out when structurally defined substrates are available in large enough quantity. The difficulty of providing sufficient amounts of material for functional studies (hampered, e.g., by the high cost of human-derived substrates and the non-trivial effort involved in their synthesis) explains why only a handful of mechanisms for the foraging of gut bacteria on human glycans have been elucidated so far ([15–24] reviewed in [13, 25]). Among them, only a few are related to the metabolism of uncultured bacteria [17, 18, 24]. Bearing in mind that uncultured species largely dominate in the microbiota, the available evidence is far from exhaustive and leaves much of the understanding of the inter-relationships between the microbiota and their host to be discovered.

Given the diversity of the human gut microbiome, activity-based screening is an attractive strategy for the functional exploration of whole microbiota. Such a functional metagenomics approach simultaneously leads to biochemical proof of function for the proteins involved and links functions to sequences encoded by the metagenome. Indeed, mining the human gut microbiome has led to the identification of CAZymes involved in the catabolism of plant-derived dietary glycans [8, 26, 27], of crude mucus with a highly complex molecular structure [28], and, more recently, in the conversion of the A- to the H-antigen of O type blood [24]. The limits of this approach are practical: automated screening of metagenomic libraries on agar plates or in micro-plates allows only up to hundreds of thousands of assays per week to be carried out [29]. In each of these conventional screening formats (liquid or solid), large amounts of substrate (~grams) and liters of culture medium are required to grow libraries of clones that are large enough to cover sufficient sequence space to detect hits (> 10^3^ Mbp) [8].

The miniaturization of screening volumes to picoliters has been achieved by compartmentalizing individual screening experiments in water-in-oil emulsion droplets that are generated and sorted in microfluidic systems at kHz rates. First used for directed enzyme evolution [30, 31], droplet microfluidics has been applied to discover microbes from environmental samples that secrete enzymes [32] or to screen metagenomic DNA libraries for enzymes performing non-natural reactions [33]. The latter study is the first (and still only) example of an application of droplet-based microfluidics in functional metagenomics. The approach involved the lysis of metagenomic *Escherichia coli* clones grown in single-cell emulsion droplets to enable the encounter of intracellular enzymes with the fluorogenic substrate. After on-chip sorting of the fluorescent droplets, the selected genes (as short metagenomic DNA fragments of ~ 2 kbp cloned into a high copy number plasmid) can be recovered efficiently [31] after de-emulsification and *E. coli* transformation. However, multi-gene clusters and operons encoding entire anabolic or catabolic pathways (such as the PULs involved in the metabolization of complex glycans [8]) cannot be identified in such short fragments. The prospect of identifying such complex systems rather than single enzymes will make the microfluidic screening of libraries with larger metagenomic DNA fragments (30 to 40 kbp) attractive. The amount of DNA screened “per assay” in a “monoclonal” droplet would be increased and a larger fraction of sequence space will be explored. Our objective was therefore to develop a workflow compatible with the recovery of large DNA fragments, typically involving low copy number fosmid vectors instead of the previously used high copy/small insert plasmid libraries [33]. We recently demonstrated selective growth in single-emulsion droplets of metagenomic clones, which are able to both internalize and to metabolize oligosaccharides [34], and these two properties are conferred to the host strain by a single PUL cloned into a fosmid [35]. In this work, we demonstrated that the metagenomic gene expression in fosmids is due to the random presence of sequences on the metagenomic DNA insert which are recognized as promoters by the *E. coli* host strain [35]. For enzyme screening, the challenge of protein expression has to be met, so various formats for protein production in droplets have been developed, e.g., yeast display [30], in vitro expression [36], or expression in *E. coli* followed by cell lysis [31]. Only the latter is compatible with the screening of a metagenomic library and would cover intracellularly as well as extracellularly expressed proteins. It was consequently used in the present work. Our workflow for enzyme discovery from metagenomes therefore starts with transformation of *E. coli* with a fosmid library*.* Efficient enzyme expression and recovery are key to the success of this approach: within droplets both phenotype and genotype were amplified by cell growth [37]. The previous metagenomic droplet screening campaign [33] had used on-chip sorting, while in this work we employed flow-cytometric sorting of water-in-oil-in-water “double emulsion” droplets [38]. The throughput of both is similar (> 10^6^ per day), but FACS instruments are widely available to non-specialist laboratories, thus avoiding the need to set up a specialized laser sorting system [39].

This set-up constitutes the first ultra-high-throughput screening pipeline for fosmid libraries but is compatible with all types of (meta)genomic libraries (i.e., containing short and large inserts) and allows the detection of intracellular and extracellular enzyme activities. The approach was validated by exploring the human glycan degrading potential of the gut mucosal microbiota, which led to the identification of key pathways involved in microbiota-host interaction in the context of IBD.

## Results

### A new pipeline for droplet-based microfluidic screening of enzymes

This study focused on the microbiota of the distal ileum mucosa, which is likely to play a role in the regulation of intestinal homeostasis and immune response [40]. Indeed, the terminal ileum is, as the rectum, an anatomical region highly predisposed to inflammation in IBD [41, 42]. In addition, the diversity of the mucosa-associated microbiota is relatively stable from the distal ileum to the rectum, in both healthy individuals and patients with IBD [43]. This analysis suggests that our sampling strategy to study the relationships between the host and mucosa-associated bacteria will cover this diversity, although the glycosylation patterns of the mucus lining the epithelium are different between the small and the large intestine, with more fucosylated, and fewer sialylated and sulfated glycans in the small intestine [44].

A metagenomic library of 20,000 fosmid *E. coli* clones, covering 0.7 Gb of metagenomic DNA, was screened by droplet microfluidics for β-N-acetylgalactosaminidase (β-GalNAcase) activity using a commercially available fluorogenic glycoside analog, resorufin-β-GalNAc, mimicking GalNAc-containing physiological substrates. Indeed, GalNAc is one of the main components of human glycans (e.g., mucus, gangliosides, milk, blood group antigens), together with fucose (Fuc), sialic acid (Neu5Ac), galactose (Gal), and glucose (Glc).

**Fig. 1:**
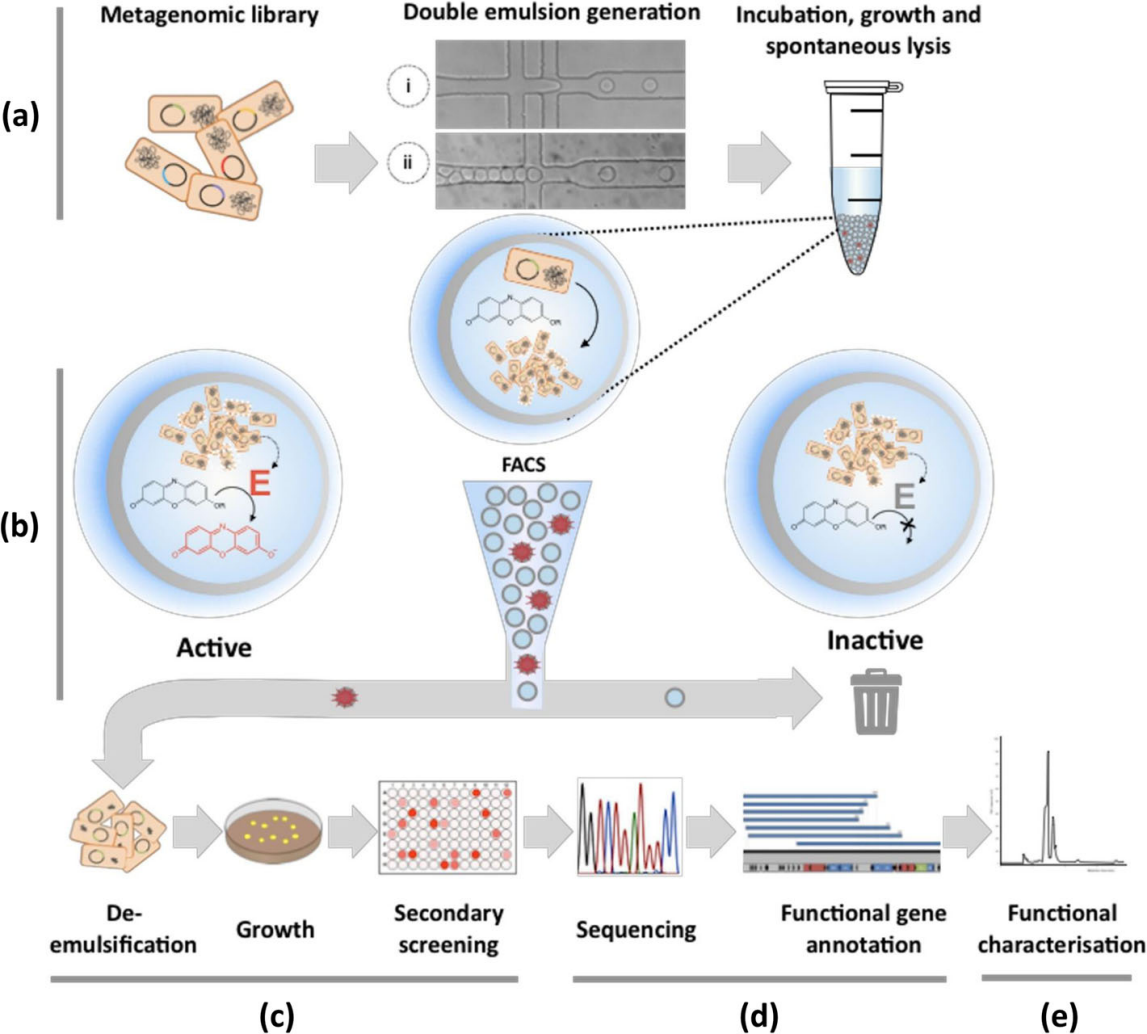
Workflow of the microfluidic picoliter droplet screening of a fosmid metagenomic library. (**a**) A library of metagenomic clones in *E. coli* was encapsulated in droplets under a Poisson distribution (*λ* = 0.35, i.e., 25% were singly compartmentalized) in the presence of a fluorogenic substrate. (**b**) After 24 to 72 h incubation, at least 6 million droplets for each condition were sorted using FACS. The droplets exceeding a set product fluorescence threshold (> 5-fold background) were pooled and demulsified (**c**) before plating the clones. On the 19,501 clones recovered from the six droplet pools after overnight growth on agar plates, a secondary screening was performed on 372 candidates per sorting condition, to quantify their hydrolytic activity at pH 8 and 9. Of 2232 assayed combinations, 144 clones exhibited 2 times the value of a negative clone and were selected for further analysis. (**d**) The metagenomic DNA inserts (ranging from 30-40 kb) were sequenced. Assignment of clonal and partial redundancies led to identification of novel human glycan utilization pathways. (**e**) The ability of the different clones to breakdown native, non-fluorogenic human glycans was finally demonstrated by HPAEC-PAD analysis

The workflow in Fig. 1 illustrates how single cells were compartmentalized into monodisperse droplets according to a Poisson distribution that gives an average of 0.35 cells per droplet to ensure mono-clonality (i.e., while 70.5% of all droplets were empty, 25% contained a single clone, and 4.5% multiple clones). To visualize enzymatic turnover in droplets, resorufin-β-GalNAc was added to the cells with a recovery medium before encapsulation. In the droplets, enzyme and substrate were brought together by the partial and spontaneous lysis of *E. coli*, where the liberated enzyme generated fluorescent product. Cell division was also necessary to maintain fully functional *E. coli* clones within each droplet and to improve chances of fosmidic DNA recovery after droplet sorting.

In order to determine the best screening conditions, three incubation times (24, 48, or 72 h before sorting) and two pHs (8 and 9) were tested to cover the likely activity range over the timescale of the experiment, which depends (i) on the kinetics of growth and spontaneous lysis in droplets, and (ii) on resorufin-substrate leakage at long incubation times. An experimental analysis was necessary, because these factors contribute in opposing directions, creating a challenge of identifying an optimum. While cell growth and survival is expected to be more efficient at pH 8 than at pH 9, the resorufin-substrate leaks faster at pH 8 than at pH 9 (Fig. S1 in Additional file 1) even though the fluorophore emits higher fluorescence at pH 9.

For each sorting condition, at least 6 million droplets were sorted by FACS, corresponding to a 90-fold oversampling coverage of the initial library diversity. A droplet fluorescence threshold of at least fivefold of the fluorescence brought about by cellular and chemical background (relative to a clone without β-GalNAcase activity) was applied in the flow cytometric droplet sorting. This tolerant threshold was deliberately chosen to avoid loss of relevant clones, given the possible phenotypic variation and variation in cell growth in droplets. The yield of droplets satisfying this criterion varied between 0.02 and 0.06%, resulting in several thousand hit droplets for each condition.

After sorting, the selected droplets were de-emulsified and plated on agar for overnight growth. This allowed us to recover the hit clones without any DNA recovery and transformation steps. Under both pH conditions, a 48 h incubation time resulted in the best clone recovery yield, specifically 179% at pH 8 and 100% at pH 9 (Table 1). These high recoveries are due to cell growth during the time of incubation. After growth, multiple occupancy of droplets by identical clones makes recovery more likely. The lower recovery yield obtained at pH 9 could be explained by the slower growth and survival rates at pH 9 compared to pH 8.

**Table 1:** Outcome of droplet-based microfluidic screening. After FACS sorting of the positive droplets and de-emulsification, cells were plated on solid agar to recover the sorted clones. For each screening condition, the activity toward resorufin-β-GalNAc of 372 of the 22,200 clones recovered on solid plates was quantified at 37 °C in 50 mM Tris/HCl, pH 8 or 9, to select those with at least twice the activity of the *E. coli* host used as negative control. Then, the clonal redundancy resulting from the bulk de-emulsification of the hit droplets was quantified by sequencing the metagenomic insert extremities for each positive clone, in order to determine the yield of different positive clones isolated from the initial metagenomic library of 20,000 clones

	Recovery yield^**a**^ (%)	Positive clones^**b**^ (%)	Different positive clones	Positive clone yield^**c**^ (‰)
Library pH 8				
24 h	146	13	14	0.7
48 h	179	8	15	0.75
72 h	94	6	9	0.45
Library pH 9				
24 h	47	9	6	0.3
48 h	100	1	2	0.1
72 h	28	1	1	0.05

^a^Recovery yield (%) = 100 × (number of clones recovered on solid plates)/(number of positive droplets)

^b^Positive clones (%) = 100 × (number of positive clones in quantitative microplate assays)/(number of clones recovered on solid plates)

^c^Positive clone yield (‰) = 100 × (number of different positive clones)/(initial library size)

Then, we further analyzed around 10% of the recovered clones, in order to establish their functional profile and to quantify false positives due to (i) the co-encapsulation of multiple cells in one droplet at the point of droplet generation (according to Poisson distribution around 4.5% of the droplets contained more than one cell at the initial incubation time) and (ii) the tolerant threshold chosen for cell sorting by FACS.

**Fig. 2:**
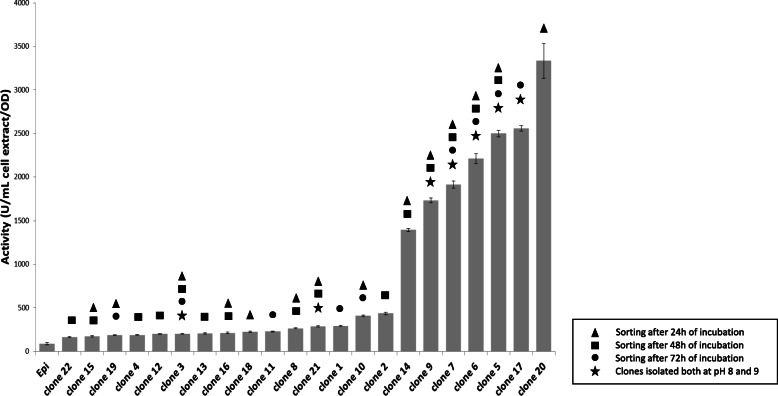
Activity of the different clones quantified in liquid medium toward resorufin-β-GalNAc. Since all the clones isolated at pH 9 were also isolated at pH 8, the activity at pH 8 (at 37 °C in 50 mMTris/HCl) of the 22 different clones identified in this study is presented in this figure. Activity was normalized with cell growth, quantified at DO_600nm_ before lysing the cells to extract the intracellular GHs. The negative clone Epi (host Epi100 *E. coli* strain) was used as negative control

The β-GalNAc-ase activity of the intracellular extracts of 2232 clones was quantified in 96-well plates (Fig. 2 and S2 in Additional file 1). The percentage of positive clones varied between 13 and 1%, decreasing with incubation time (from 24 to 72 h) at both pHs (Table 1). The following considerations rule out the fact that the low percentage of positive clones might be due to the loss of the fosmid or of the insert DNA during the screening process. The use of a chloramphenicol resistance marker instead of ampicillin militates against the survival of cells without fosmid in the proximity of the β-lactamase secreting ones. In this study, chloramphenicol was present in the culture medium in all steps of the pipeline, maintaining selection pressure for preservation of a fosmid throughout. Additionally, the vector pCC1FOS has been shown to be stable in the EPI100 *E. coli* strain after three replications and 3 days of growth on solid plates without chloramphenicol (unpublished results). Finally, F replicon-based, single-copy fosmids such as pCC1FOS are known to stably maintain insert DNA [45]. Thus, the cytometric detection of such a high number of false positives is more likely due to (i) the tolerant sorting threshold that was deliberately chosen to avoid missing positive droplets; and (ii) the sorting of weakly active clones (based on nanomolar FACS sensitivity) with high growth rate in droplets, which were not selected after the secondary activity screening in microplates, normalized for cell growth. Besides, despite the observed leakage of resorufin-β-GalNAc (at pH 8 beyond 15 h of incubation, Fig. S1), a large number of genuine hits could be validated by re-screening, suggesting that practical concerns about leakage can be overcome in this way, even for long incubation times.

The success of the strategy in identifying a total of 144 active clones demonstrates that at least one cell survives the incubation period in the droplet and the demulsification step, and that these cells can be recovered by growth. If more cells are recovered or if identical sequences are identified as a result of screening at least 90 identical copies of each library member, this process results in clonal redundancy after cell recovery from the demulsification medium. Thus, in order to avoid the complete sequencing of the same metagenomic clone several times over, we first determined clonal redundancy by N- and C-terminal sequencing of all the active clones identified after activity quantification (Table S1 in Additional file 1). We considered sequences to be identical when N- and C-termini were identical between two clones (on average 600 bp, which corresponds to the length of the sequences obtained by Sanger sequencing from the vector extremities). We validated this hypothesis by complete sequencing of two DNA inserts with N- and C-termini identical to those of clone 22 and clone 8. These sequences were strictly identical to those of clone 22 and clone 8, respectively.

For each sorting experiment, between 1 and 15, different clones were obtained, giving an overall clonal hit yield between 0.05 and 0.75‰. Comparison of timescales (Tables 1 and S1) shows that some clones go amiss for longer incubation (correlated to the decrease of the recovery yield as a result of high lysis level at 72 h, in particular at pH 9). Under the best screening condition (pH 8, 48 h incubation), we sorted 1.9 k droplets by FACS, recovered 3569 colonies, picked 372 of these for the secondary screen, in which 31 had high activity and were sequenced to reveal 15 unique hits. This result was obtained under conditions that we presume to represent the optimal balance between *E. coli* growth and lysis rates in droplets, enzyme activity at basic pH, and substrate/product leakage during the long incubation time required for spontaneous cell lysis (48 h incubation at pH 8). The highest hit rate was three times lower than that obtained by automated screening of the same library for β-GalNAcase activity on solid plate (2.3‰, from [28]). Nevertheless, sequence redundancy had not been assessed in this previous study, which might have resulted in over-estimation of the hit rate. In addition, this previous conventional screening effort took 30 times longer and required 5000 times more substrate than our microfluidics-based workflow to explore the same sequence space, and this, without the 90 replicates for each library member that we screened in this work.

In order to establish that the selection outcome was consistent with the substrate used, and to detect complementary activities due to enzyme promiscuity toward hexosaminoglycosides or to the presence of several CAZyme encoding genes clustered in the selected metagenomic loci, we compared the activity of the 22 different active clones selected by droplet-microfluidics against 5-bromo-4-chloro-3-indolyl-β-GalNAc (X-β-GalNAc) and 5-bromo-4-chloro-3-indolyl-β-GlcNAc (X-β-GlcNAc). These chromogenic substrates have been previously used for conventional screening of this metagenomic library on solid plates. Interestingly, eight of the clones were active both on X-β-GalNAc and X-β-GlcNAc, while more than half of the different clones were not active on either of the tested X-substrates (Table S2 in Additional file 1). These results clearly highlight that the CAZyme activity is affected by the structure of the chromogenic/fluorogenic aglycone.

### Identification of human glycan degradation pathways

**Fig. 3:**
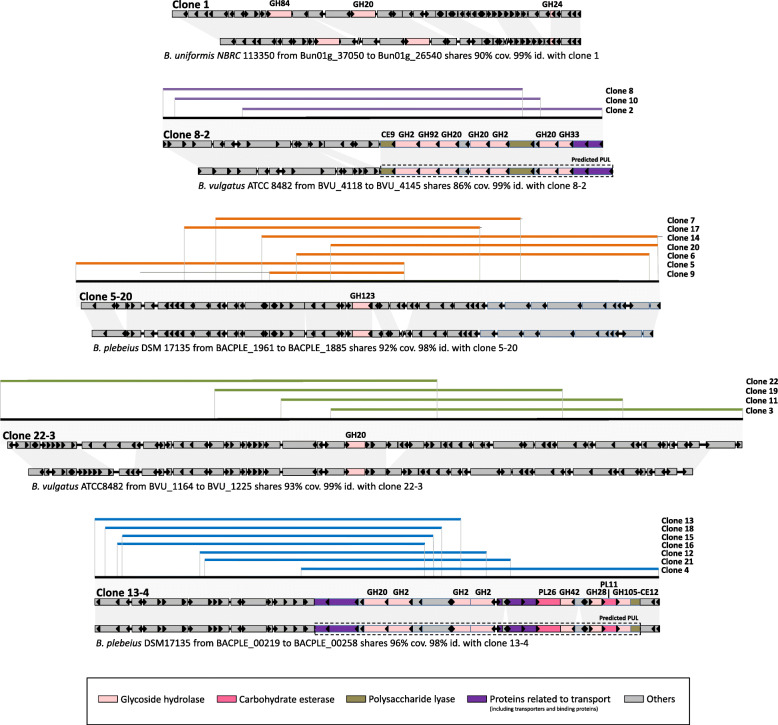
Gene organization and partial sequence redundancy of the hit clones. The best synteny with referenced genomes is indicated with gray bars

The metagenomic DNA from the total of 22 different clones was sequenced using high sequencing depth (100X), ensuring reliable sequence assembly. For each clone, one contig sized between 32,148 and 43,348 bp was obtained. Among the 641 annotated genes, we identified 61 CAZymes including 56 GH family members, three carbohydrate esterases (CE), and two polysaccharide lyases (PL). All of the clones harbored at least one GH sequence assigned to the GH20 and GH123 CAZy families, which are likely responsible for the screened activity (Table S3 in Additional file 2). However, SDS-PAGE analysis of the *E. coli* cell extracts did not allow us to highlight overproduced proteins (Fig. S3 in Additional file 1), indicating that the specific activity of the CAZymes is sufficiently high to detect significant activity levels (Figs. 2, S2 and S3), despite a low yield of recombinant protein production. In such *E. coli* fosmid clones, only some genes of the cloned loci are expressed spuriously, likely due to the presence within the metagenomic sequence of σ70 promoter sequences for *E. coli* [35, 46, 47]. In these metagenomic sequences, one CAZyme was found every 13 kb, which is 3.5 times more frequent than in the randomly sequenced human gut metagenome [48]. Except for clone 1 that displays a unique sequence, partial redundancies were observed for all the contigs (Fig. 3), with 100% sequence identity on 7867 to 35,872 bp. This allowed us to define five metagenomic loci by contig assembly (with size between 46,158 and 72,080 bp). In total, 22 different CAZyme sequences were identified. Repetition of one of the experiments displayed in Table 1 (24 h incubation, pH 8) led to nearly identical outcomes (Tables S1 and S4 in Additional file 1): 12 different hit clones instead of 14 were found, and no other CAZyme sequence was identified (Tables S1 and S3). This exquisite screening reproducibility demonstrates the high-quality of the droplet assays and the beneficial effect of the 90-fold oversampling that is feasible because of the ultrahigh throughout possible in droplet screening.

**Fig. 4:**
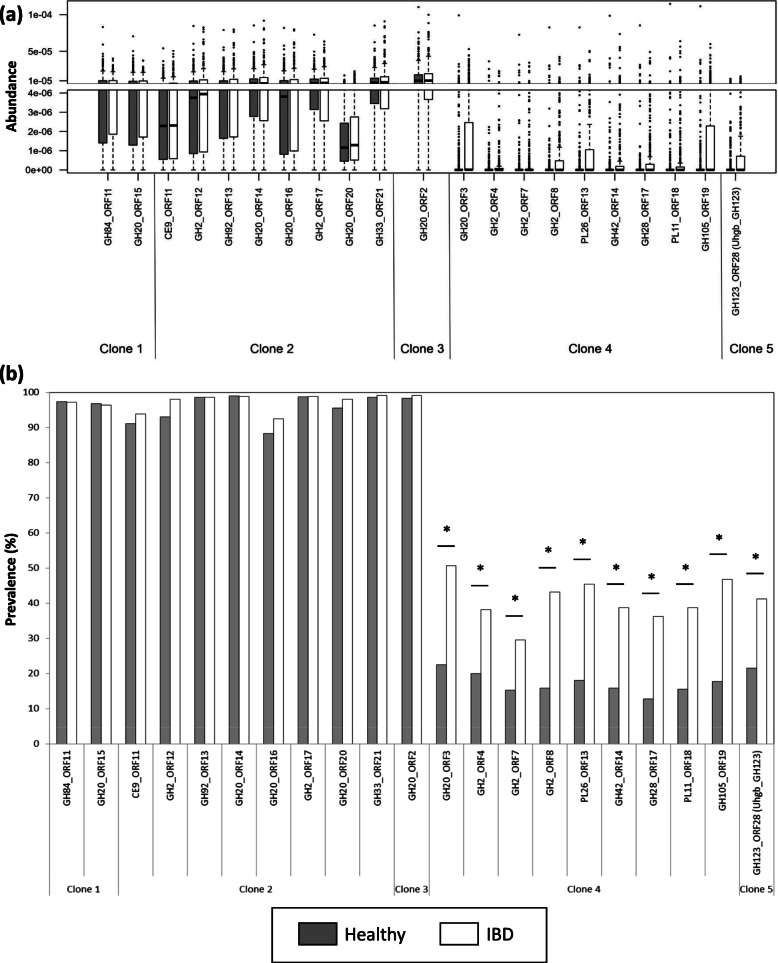
Abundance and prevalence in the human gut microbiome of the newly discovered CAZymes encoding genes. Gene abundance (**a**, relative abundance of genes in samples that contain them) and prevalence (**b**, percentage of subjects whose microbiome contains the target genes) were determined in the gut metagenome of 760 European subjects including 401 healthy and 359 IBD affected individuals [51]. Asterisks indicate significantly different prevalence between the two clinical status groups (*p* value < 10^−5^)

In order to analyze the biological role of the identified metagenomic loci in the human gut, further in-depth sequence analysis was performed, with activity profiling on β-GalNAc and β-GlcNAc containing human gangliosides and human milk oligosaccharides that share structural similarity with intestinal mucins (Table 2, resulting from HPAEC-PAD analyses presented in Fig. S4 in Additional file 1). These oligosaccharides are devoid of the chromophore or fluorophore which could affect activity, and represent physiological substrates that mucosal bacteria encounter in their habitat. Five clones (clone 1, clone 2, clone 3, clone 4, and clone 5) were selected to represent the diversity of all the CAZymes identified after the sequencing step. Selection was based on the presence within their sequence of the highest number of CAZyme encoding genes, which could be involved in the clone phenotype (clone 2 and clone 4), or on their high contig length and activity level on the screening substrate (clone 3 and clone 5). These representative metagenomic loci were assigned to the *Bacteroides* genus, with marked syntenies with genomes of human gut cultivated strains (Fig. 3), highlighting likely horizontal gene and loci transfers between gut bacteria. Indeed, horizontal gene transfers have largely been demonstrated within the human gut microbiome, as impacting functions linked to glycan utilization [8, 49, 50]. In addition, the presence of mobile elements, known as main agents of horizontal gene transfer, in some clones (clone 1, clone 4, clone 5, clone 6, clone 7, clone 9, clone 14, clone 17, clone 20, and clone 21) could underline this phenomenon. Homologous sequences of all the CAZyme encoding genes identified in this study, from the clone 1, clone 2, clone 3, clone 4, and clone 5 loci, were found in both gene catalogs of the human gut microbiome [51, 52]. The gene abundance counting in the human gut microbiome of 760 individuals, from a European cohort comprising healthy individuals and IBD patients [51], revealed two categories of sequences (Fig. 4).

**Table 2:**
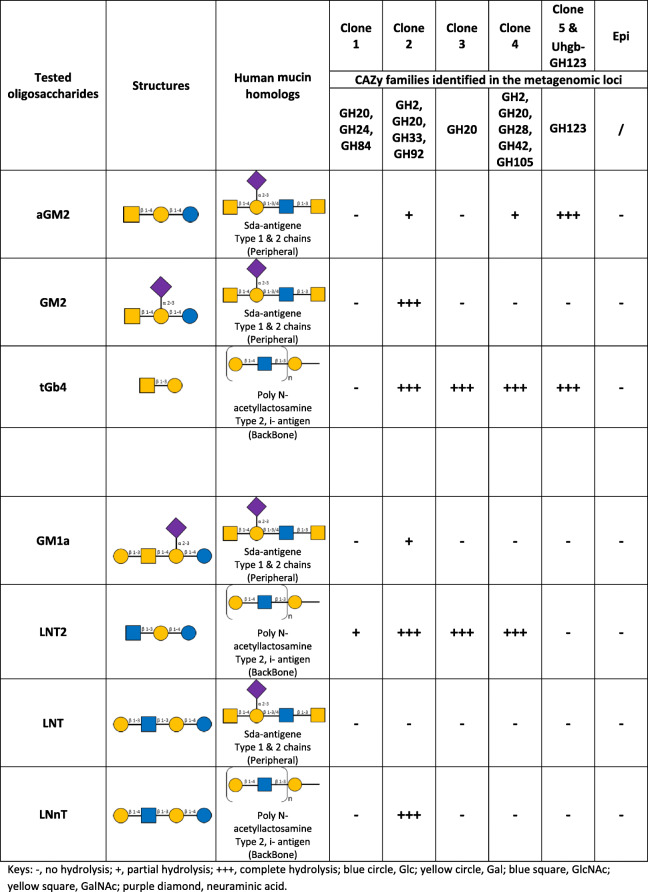
Summary of the ability of the hit clones to hydrolyze human glycans. Cell extracts, containing one (clone 3 and clone 5) or several GHs (clone 1, clone 2, and clone 4), were incubated with 2 mM host-derived oligosaccharides for 24 h at 37 °C in 50 mM sodium phosphate buffer pH 7, before analysis by HPAEC-PAD (detailed in Fig. S4). The tested oligosaccharides, which share structural homologies with intestinal mucins, were the aGM2, GM2, tGB4, and GM1a ganglioside sugars, and the HMOs lacto-N-triose (LNT2), lacto-N-tetraose (LNT), lacto-N-neotetraose (LNnT). The *E. coli* Epi100 screening strain constitutes the negative control. The purified Uhgb_GH123 enzyme isolated in clone 5 was tested in the same conditions on these substrates, using 2 μM of enzyme.

Keys: -, no hydrolysis; +, partial hydrolysis; +++, complete hydrolysis; blue circle, Glc; yellow circle, Gal; blue square, GlcNAc; yellow square, GalNAc; purple diamond, neuraminic acid

#### Highly abundant host glycan degradation pathways in the gut microbiome

The clone 1, clone 2, and clone 3 genes encoding CAZymes are highly abundant and prevalent in the human gut microbiome, regardless of the medical status of the individuals (Fig. 4). Indeed, their average relative abundance in the human gut metagenome is in the same range as that of the most abundant genes of the catalog [51], and these genes were found in more than 85% of the individuals. Clone 1 shares synteny with the genome of *Bacteroides uniformis* NBRC 113350, and the GH20 shares more than 99% identity (100% coverage) with its GH20 Bun01g_36790 (GenBank ID: BBK89309.1). In addition to the GH20, the clone 1 contig also contains GH84 and GH24 sequences. Both the GH20 and GH84 families contain β-N-acetylhexosaminidase members, in particular β-N-acetylglucosaminidases, while characterized members of family GH24 are all lysozymes. Even if the clone 1 extract exhibits a weak activity on the screening substrate resorufin-β-GalNAc, it produces much more β-GlcNAcase than β-GalNAcase activity, being active only on X-β-GlcNAc and on the human milk oligosaccharide LNT2 but not on GalNAc containing human glycans (Fig. S4a and Table 2). This activity profile is consistent with a role of the GH20, the GH84, or both enzymes in GlcNAc/GalNAc breakdown.

The clone 2 and clone 3 sequences both present synteny with two loci from *Bacteroides vulgatus* ATCC 8482. Clone 2 includes the predicted PUL from BVU_4133 to BVU_4145 referred in the PUL database (PULDB, [53]), which contains six GH-encoding genes (one GH33 and GH92, two GH2, and three GH20). This CAZyme-rich locus has also recently been isolated by conventional screening of the same library [28] (clone 14 N11, corresponding to clone 10 in the present study, of which the sequence is largely covered by that of clone 2), and proved to be involved in the alteration of the colon mucus structure using lectin binding assay. The present PUL shows a close gene organization to the recently characterized N-glycan PUL from BT0451 to BT0461 with relatively high sequence identity between homologs from 64 to 79% (with 96-100% coverage) [23] (Table S3). Here, we refined the mechanisms of degradation of this complex mucus matrix by demonstrating that this locus is involved in the hydrolysis of a large panel of human glycans of defined structures (Fig. S4a-f). Clone 2 indeed hydrolyzed terminal β-(1,3)-linked GlcNAc and GalNAc motifs regardless of the linkage β-(1,4) or β-(1,3), and also β-(1,4)-linked Gal residues, but not β-(1,3) linked ones. In particular, the GM2 ganglioside was completely hydrolyzed, probably due to the presence of the GH33 enzyme which might be responsible for the hydrolysis of the neuraminic acid residue, while GH20 and GH2 might be involved in the hydrolysis of GlcNAc/GalNAc and of Glc/Gal, respectively. These results are in agreement with the activities observed for the homologous enzymes individually characterized by Briliute et al. [23]. The second locus assigned to *B. vulgatus*, namely the clone 3 sequence, contains only one GH20 encoding gene. This enzyme could not have been identified by conventional screening, clone 3 (and the other clones containing this locus) being inactive on X-GalNAc or GlcNAc substrates (Table S2). In addition, as for clone 1 and clone 5, this locus could not have been retrieved by sequence-based mining of the PULDB, in which it is not listed since it does not include any SusD encoding gene. However, here, we show that this highly abundant sequence may play a key role in the bacterial degradation of host glycans, especially those containing terminal β-(1,3) linked GlcNAc (like LNT2), and also terminal β-(1,3) linked GalNAc (like tGB4) (Fig. S4a and c, Table 2). The GH20 gene of clone 3 probably encodes a secreted protein or a lipoprotein, since signal peptidase I (SpI) and II (SpII) cleavage sites were predicted in its sequence. If it is a lipoprotein, it is likely attached to the outer membrane, since there is no aspartic acid in position +2 after the SpII cleavage site. The promiscuity of this GH20 toward β-linked hexosaminides, and its cellular location, probably confer an ecological advantage to the species producing it in the mucosal habitat by allowing it to forage on a large panel of human glycans. This could explain the high prevalence and abundance of this sequence in the microbiome.

#### Highly prevalent pathways in inflammatory context

The second group of sequences contains the clone 4 and clone 5 contigs. Except for the two highly prevalent genes encoding mobile elements (clone 4_ORF10 and clone 5_ORF3, both homologs to gene DLM022_GL0145524 of the catalog which is present in 86% of the subjects) distributed among intestinal bacteria (principally in *Bacteroides*, *Prevotella*, *Odoribacter*, *Porphyromonas*, *Alistipes* spp., and the *Mucinovorans* pathogens), the clone 4 and clone 5 sequences are much less prevalent in the gut microbiome than those of clones 1-3 (Fig. 4). Nevertheless, many genes of the clone 4 and clone 5 loci, including GalNAc active enzymes, are significantly more prevalent in IBD affected individuals (*p* value < 10^−5^). They both share high synteny with *Bacteroides plebeius* DSM 17135 genome. Clone 4 contains nine CAZyme-encoding sequences (three GH2, GH20, GH42, GH28, one bifunctional GH105-CE12, PL11, and PL26), corresponding to the predicted PUL from BACPLE_00240 to BACPLE_00256 of the PULDB (Fig. 3), to which no substrate had been associated yet [53]. The combination of CAZy modules in this PUL is very similar to those of PULs from *Bacteroides thetaiotaomicron* VPI-5482 (from BT4145 to BT4183) and *Bacteroides xylanisolvens* XB1A (from BXY_32120 to BXY_32390) which target type I rhamnogalacturonan, a component of pectins [54, 55]. In the human gut, the clone 4 locus thus probably targets this dietary glycan. The β-hexosaminadase (Fig. S4a-c) activities that we detected for clone 4 may be due to substrate promiscuity of one or several of the produced CAZymes, since the presence of GlcNAc or GalNAc in pectins has never been shown. Besides, the clone 5 locus, which contains only one GH123 sequence, is not listed in the PULDB since it does not include any SusD encoding gene. In addition, all the clones producing this GH123, which were the most frequently retrieved from this screening (at all incubation times and both pH, Fig. 3 and Table S1), were inactive on X-GalNAc or X-GlcNAc (Table S2) and could thus not have been identified either by conventional screening. Interestingly, the high prevalence of this GH encoding gene correlates with IBD incidence, since it is found to be twice as prevalent in the IBD affected individuals (42% of the IBD subjects, versus 21% of the healthy ones). The specificity of this key enzyme was thus further investigated at the molecular level.

### Uhgb_GH123, a prevalent ganglioside-degrading enzyme in the gut microbiome of IBD patients

The clone 5 sequence encoding the GH123 enzyme (named Uhgb_GH123) shares 100% identity (and full sequence coverage) with BACPLE_01933 (GenBank ID: WP_007561385.1), a gene encoding a DUF4091 domain-containing protein from *B. plebeius* DSM 17135. Its sequence similarity with the three characterized GH123 members is low, with 53% identity with *Bv*GH123 from *B. vulgatus* ATCC 8482 [56], 35% with NgaP from *Paenibacillus* sp. TS12 [57], and 30% with *Cp*GH123 from *Clostridium perfringens* ATCC 13124 [58]. We searched for *Bv*GH123, NgaP, and *Cp*GH123 sequences in the microbiome, in order to compare their prevalence with that of Uhgb_GH123. This analysis revealed that no NgaP homolog sequence was found in the gene catalogs of the human gut microbiome and that *Cp*123 encoding gene is found in only 4% of the subjects (Fig. S5 in Additional file 1). By contrast, we show that the *Bv*GH123 encoding gene is highly prevalent (88% of the subjects) and abundant, regardless of the health status of the individuals, confirming the prominence of *B. vulgatus* in the gut. Only the Uhgb_GH123 sequence is more prevalent in the IBD cohort (42% versus 21% of the healthy individuals), highlighting its possible implication in gut inflammation. This gene was also abundant in the metagenomic DNA sampled in the present study, since it was found in seven of the 22 different clones identified, representing 21% and 55% of the positives clones retrieved at pH 8 and pH 9, respectively (Table S1). This might be due to the fact that this sample was issued from a patient with suspected colon cancer, a pathology resulting from chronic intestinal inflammation.

In order to characterize Uhgb_GH123 at the molecular level, its encoding gene was subcloned to produce a soluble protein with a N-terminal (His)_6_ purification tag. The specificity of the purified enzyme was characterized by determining its kinetic parameters on various synthetic *p*-nitrophenyl glycosides. The purified Uhgb_GH123 was only active on *p*NP-β-D-GalNAc and *p*NP-β-D-GlcNAc. No activity was observed on *p*NP-α-D-GalNAc, *p*NP-α-D-GlcNAc, *p*NP-α-D-Glc, *p*NP-β-D-Glc, *p*NP-α-D-Man, and *p*NP-β-D-Man. Uhgb_GH123 displays a pH optimum of 5.5, a temperature optimum of 30 °C, and a 213-fold higher catalytic efficiency on *p*NP-β-D-GalNAc than on *p*NP-β-D-GlcNAc (Table S5 in Additional file 1). This preference for β-galactosaminides versus β-glucosaminides is also a trait of *Bv*GH123 [56], indicating that these two enzymes might act on the same physiological substrates. However, the ability of *Bv*GH123 to hydrolyze human glycans of defined structures has never been studied.

In order to identify the physiological substrate of Uhgb_GH123, its specificity toward host-derived glycans harboring β-linked N-D-acetylgalactosamine or β-linked N-D-acetylglucosamine residues was determined using HPAEC-PAD analysis of hydrolysis products (Table 2, Fig. S4g). Like the cell extract of clone 5 (Fig. S4a-c), which only contains this GH, Uhgb_GH123 cleaved the terminal β-D-GalNAc residue of aGM2 and tGb4 substrates, with no significant preference for β-(1,4) or β-(1,3)-GalNAc linkages. GM2 was resistant to enzymatic hydrolysis, indicating that the presence of the neuraminic acid residue prevents accommodation of sialylated substrates in the enzyme active site.

## Discussion

### Ultra-high throughput sorting of intracellular enzymes

In this study, we have shown the screening of 20,000 library members with 90-fold repetition, covering 0.7 Mb of metagenomic DNA (the equivalent of 200 bacterial genomes) in 4 min. This is 2600-fold faster than an automated colony screen. This extensive screening experiment (under six different conditions) was possible with less than 0.01 mg of fluorogenic substrate (at a cost of $0.1), while an automated colony screen or a robotic liquid handling experiment would have consumed 900-fold (assuming 7.2 mg for the 9 large screening plates required to screen 20,000 clones [29]) and 2000-fold more substrate (assuming 100 μl in each well of a 348-well plate), respectively. In addition, by using microfluidics, it was possible to screen each clone on average 90 times (resulting in 1.8 million assays), which contributed to excellent reproducibility. Disregarding the cost of instruments (~ $39 k for a microfluidic set-up vs $300-750 k for an automated robotic system) the consumable costs required to perform 1.8 million assays were $346 in our microfluidic assay, compared to ~ $112 k using a robotic system with 384-well plates (Table S6 in Additional file 1). By using this efficient technology, success is more likely, as genuine hits are bound to show up when tested multiple times, even if the detected activity is close to background. This versatile approach could also be used with any other chromogenic/fluorogenic substrates employing analogous sorting strategies, such as absorbance-activated droplet sorting (AADS) [59]. Here, we used resorufin-β-GalNAc to mimic the structure of human-derived glycosides. The only other commercial fluorogenic substrates containing residues highly abundant in human glycans, 4-methylumbelliferyl-α-Neu5Ac and 4-methylumbelliferyl-α-Fuc, were not compatible with our droplet-based assay, in which cell growth, lysis and substrate hydrolysis were performed in one single step. Indeed, in order to detect the signal, the fluorescence of 4-methylumbelliferyl would have to be 100-fold more intense, which could be achieved by adding a pH 10.7 buffer [60]. However, this would have prevented cell growth and enzyme activity, since most CAZymes from the human gut microbiome are inactive above pH 9 [8]. To avoid the use of chemically modified substrates that could result in screening bias, coupled assays involving unlabeled substrates and various pH ranges could also be developed.

In practice, our approach is based on *E. coli* division and partial spontaneous lysis in droplets, allowing the release of intracellular enzymes into the extracellular medium, where they would encounter the substrate. In addition, maintaining living cells in the same droplet as lysed cells allowed their function as a marker for the tested clone that can be recovered efficiently after amplification of recovered cells by growth. We demonstrate that now, multiple intracellular enzymatic activities produced from long DNA (meta)genomic loci, cloned into low copy vectors (such as fosmids), can be screened by droplet-microfluidics, widening the possibilities for functional library screening at ultrahigh-throughput beyond previous campaigns using high copy plasmids. This set-up will make it possible to employ a variety of vectors without requiring a PCR step for recovery sequence information. PCR recovery of fosmids has indeed never been performed in activity-based metagenomics, although it is theoretically possible to amplify several kbp by a rolling-circle process [61]. After overcoming the previous limiting requirement of high copy plasmids for recovery [33], new applications can be imagined, since most metagenomic resources are sequences cloned into fosmid *E. coli* libraries [62, 63]. Likewise, bacterial artificial chromosomes (BACs) or plasmids with low DNA copy number should also become amenable to droplet screening. If growth can be spatially adjusted, an extension of this work flow to any expression host [64] is also conceivable. Finally, in addition to single enzyme screening, ultra-high-throughput discovery and combinatorial engineering of complex metabolic pathways is now practically possible.

The new workflow is extremely economical and may fundamentally shift the effort in metagenomic exploration from screening (which is expensive in terms of time, equipment, handling personnel, and reagents) to a format that yields experimental hits in an afternoon. Implementation of such a procedure is straightforward—requiring simple use of pumps for droplet formation—and replaces the delicate handling of a sorting chip by a commercially mature instrument, a flow cytometer. Access to this technology should therefore allow a much wider circle of non-specialist users to engage in functional metagenomics or in metabolic pathway engineering. Instead of experimental screening, bioinformatic analyses and confirmation of hits by detailed studies may henceforth become the rate-limiting steps in a discovery workflow. The successful identification of 22 human-glycan degrading clones in this proof of concept study underlines the validity of our approach, in particular the ability to screen multiple copies of each library member separately, which provides a very robust and reliable assay. Functional information was readily obtained despite low expression of fosmid DNA and its difficult recovery—with both problems solved by cell growth in droplets. The remaining practical obstacles can also be solved: for example, the clonal redundancy was partially a consequence of having to grow cells in droplets (to increase enzymatic turnover signal and for better recovery), but this complication is easily solved by sequence analysis, by a secondary screen to eliminate clones with the same functional profile, or even by droplet isolation in microtiter plates, which can be performed by FACS.

### Capture and characterization of human glycan degradation pathways

The quality of the data generated at ultra-high throughput allowed us to rapidly gain insight into the functional potential of the mucosal microbiota, and to decipher, from the ecosystemic to the molecular levels, aspects of their relationships with the host.

#### Revealing the role of Bacteroides species in human glycan degradation and correlation to gut inflammation

The taxonomic annotation of the large metagenomic loci we were able to capture was based on that of 30 genes per locus on average. This annotation is thus much more accurate than that of small contigs containing only one gene, like those obtained by sequence-based metagenomics [51].

In this study, all the identified metagenomic loci were assigned to the *Bacteroides* genus. However, the screened metagenomic library contains DNA fragments from other genera. Analysis of the 16S rDNA from the metagenomic DNA before cloning indeed demonstrated that the sampled mucosa-associated microbiota displays a phylogenetic make up resembling that of ileum and colonic samples [65], with 52% *Firmicutes* (in particular *Ruminococcus bromii*, *Faecalibacterium prausnitzii*, *Ruminococcus lactaris*, *Ruminococcus gnavus*, *Roseburia intestinalis*, and *Eubacterium* spp. as dominant OTUs), 40% *Bacteroidetes* (in particular *Bacteroides vulgatus*, *Bacteroides stercoris*, *Prevotella* spp. oral, and *Prevotella ruminicola*), and 8% others. Besides, we have previously shown that *E. coli* is able to successfully express genes and produce functional proteins from Gram positive and Gram negative gut bacteria from various genera [8]. The particular library used in this study was screened for the degradation of dietary fibers (prebiotics or plant cell wall polysaccharides), and the sequences were assigned either to *Bacteroidetes* (53% of the sequenced clones), or *Firmicutes* (47%) [27, 66], while most of the hit clones belong to *Bacteroidetes* (97%) when the screening targets the degradation of host glycans ([28]; present study). This is in agreement with the ability of *Bacteroidetes* to forage on mucin glycans and/or HMOs [1, 54, 67–72]. Nevertheless, this comparison does not mean that only *Bacteroides* species can degrade host glycans containing β-linked GalNAc residues, nor that known host-glycan degrading species from other genera (like *Akkermansia muciniphila* [73, 74] or *Ruminococcus gnavus* [75]) are absent in the mucosal ileal microbiota. For example, we detected the presence of *Ruminococcus gnavus* in our starting sample, but no sequence from this particular species was recovered after screening. This paradoxical observation might be due to the cloning bias described for large-insert metagenomic libraries, resulting in under representation of species with low G + C content, such as most *Firmicutes* [63]. In addition, we have previously shown that metagenomic gene expression in fosmid *E. coli* clones is spurious [35], as explained above. Finally, some human glycan degrading enzymes might not have been identified under our screening conditions. Indeed, some types of CAZymes are inactive toward chemically modified sugars [76]. Additionally, the intestinal pH ranges between 5 and 7 [77], suggesting that most enzymes from the microbiome would have pH optima below neutral.

Here, we highlighted four loci that share significant similarity with *B. vulgatus* ATCC8482 and *B. plebeius* DSM17135 loci*. Bacteroides vulgatus* is one of the most abundant bacterial species in the human gut. It was shown to be overabundant in several IBD studies [78–82], and described as a pathobiont due to its ability to invade gut epithelial cells and induce IBD in response to host and/or environmental triggers [83, 84]. *Bacteroides plebeius* does not seem to have the same impact on the intestinal mucosa in the inflammatory context. Mondot et al. (2015) have shown that its abundance may be associated with homeostasis and remission in Crohn’s disease patients after ileocolonic resection [85].

IBD are characterized by an unbalanced composition of the intestinal microbiota, termed “dysbiosis” [2, 86], and by an uncontrolled inflammatory response to luminal content [87]. In addition, in IBD the intestinal barrier function is severely impaired, with a thinner outer mucus barrier [88], and the layer closest to the epithelium is invaded by commensal bacteria or pathogens, including adherent-invasive *E. coli* (AIEC) in the ileum form of Crohn’s disease [41, 89]. Several species associated with IBD, like *Ruminococcus gnavus*, are efficient mucus degraders [20, 90]. However, despite this evidence, the causality links between dysbiosis, alteration of the mucus layer by gut bacteria and chronic inflammation in IBD, are not yet fully established, especially in humans [42]. The present in vitro results allow us to extend the still short list of human glycan degraders, and to revisit the catabolic potential of *B. vulgatus* and *B. plebeius*. Moreover, the high prevalence and abundance of the metagenomic loci identified here indicate that the enzymes they encode are key actors in functioning microbiota. This result also underlines the huge gap existing between our knowledge of glycan degrading pathways in microbial ecosystems and their real diversity. This is especially true for the human gut bacterial pathways targeting host glycans, which still constitute the dark matter of the microbiome, having been much less studied than plant polysaccharide degrading pathways.

#### Ganglioside degradation and implication for human health

To date, only a few pathways of ganglioside degradation by commensal human intestinal bacteria have been described [91]. In family GH123, the only characterized ganglioside degrading enzymes are *Cp*GH123 (from the human pathogen *C. perfringens* ATCC 13124) [58], and *Pa*GH123 (from the soil bacterium *Paenibacillus* sp. TS12) [57], which were shown to hydrolyze the GA2 (GalNAc-β1,4-Gal-β1,4-Glc-β1,1-Ceramide) and Gb4 (GalNAc-β1,3-Gal-α1,4-Gal-β1,4-Glc-β1,1-Ceramide) structures. *Bv*GH123 (from the human gut bacterium *B. vulgatus* ATCC 8483) was only tested on the chromogenic substrates X-β-GalNAc and X-β-GlcNAc. These three enzymes are the only characterized members of the GH123 family up to date. In the present study, we identified and characterized a new GH123 member, named Uhgb_GH123. We demonstrated that this enzyme is able to hydrolyze different gangliosides, including aGM2 and tGb4, which have structures similar to the GA2 and Gb4 ceramides, respectively.

Gangliosides are glycolipids that play major roles in signal transduction, cell adhesion, antigen recognition, and protein trafficking. In humans, they mostly occur in brain tissues [92] but they are also present in extraneural locations (spleen, liver, intestinal, and urinary tract epithelial cells). They are also components of foods such as human breast milk and dairy products. Gangliosides in the intestinal mucosa play a crucial role by preventing inflammation, susceptibility to pathogens and loss of gut barrier integrity, and accelerated ganglioside catabolism contributes to pathogenesis of IBD [93, 94]. These observations are in accordance with the fact that we found the Uhgb_GH123 encoding gene significantly more prevalent in the microbiome of IBD patients than in that of the healthy individuals. The Uhgb_GH123 sequence is not a gene biomarker by itself, since it can be found in low abundance in some healthy individuals. In addition, we do not know whether this high prevalence in IBD is a cause or merely a consequence of the disease. Nevertheless, the high prevalence in IBD of the genes encoding this multispecific ganglioside degrading enzyme and its homologs suggests that they may play a role in the inflammatory process. The results of our in vitro metagenomic study will thus have to be held against those of in vivo studies. The objective of these analyses will be to further decipher the role of Uhgb_GH123 and the other human glycan degrading enzymes identified here in the breakdown of intestinal gangliosides and mucins and, more generally, in the host-microbiota interaction in the context of IBD.

## Conclusion

Activity-based functional metagenomics is a powerful approach to boost the discovery of functions in microbiomes. However, the conventional methods are limited by the large amounts of substrate and culture medium that are required for automated screening on agar plates or in micro-plates. Here, we developed a generic droplet microfluidics-based method to speed up and miniaturize the screening process by several orders of magnitude, while being compatible with any type of library and metagenomic DNA size. The proof of concept was established by mining the mucus-associated human gut microbiome for host-glycan degrading enzymes, highlighting novel pathways of metabolization of human glycans by gut bacteria, in particular pathobionts. These results offer opportunities to facilitate future holobiomics studies, to achieve a better understanding of the host-microbiota interactions.

## Methods

### Metagenomic library construction

The metagenomic library has been constructed in a previous study [26]. Briefly, a healthy distal part of the ileum was collected from a 51-year-old male patient undergoing colonoscopy and surgery for suspected lower colon cancer, after he had been submitted to a cleansing preparation. The patient did not receive any antibiotics or other drugs during 6 months before sampling. A segment of 2 cm^2^ was obtained from a healthy zone, immediately frozen and kept at −80 °C until processing. The ileal mucosa was scraped and an enriched bacterial fraction was recovered by applying the method described in Courtois et al. [95]. The metagenomic DNA was extracted as described by Tasse et al. [8]. A 16S rDNA library was performed using S-D-Bact-0350F-a-S-20 (*E. coli* numbering) CT CCTA CGG GAG GCA GCA GT [96] and 1392R GC G GTG TGT AC A AG A/G CCC [97]. Metagenomic DNA fragments sizing from 30 to 40 kb were isolated as described by Tasse et al. [8], and cloned into the pCC1FOS fosmid (Epicenter Technologies). EPI100 *E. coli* cells were then transfected to obtain a library of 20,000 clones covering 800 Mb of metagenomic sequences. The library was stored in micro-plates to be screened either by conventional automated methods or by using droplet microfluidics. To do so, recombinant clones were transferred to 384-well microtiter plates containing a Luria-Bertani (LB) medium, supplemented with 12.5 μg/mL chloramphenicol and 8% (w/v) glycerol. After 22 h of growth at 37 °C, the plates were frozen and stored at −80 °C.

### Microfluidics-based screening

#### Materials

The materials used were purchased from Sigma Aldrich unless otherwise indicated. Fluorinated oils HFE-7500 were purchased from 3 M Novec and the 008-Fluorosurfactant from RAN biotechnologies. Resorufin-N-acetyl-β-D-galactosamine was purchased from Markergene (M1037), EDTA-free protease inhibitor from Roche (11873580001), Lysonase (71230), and BugBuster (70921-4) from Millipore.

#### Device fabrication

All microfluidic devices for monodisperse emulsion formation were produced using soft lithography as previously detailed [98]*.* A flow-focusing design was used (20 μm intersection width and 15 μm channel height) with three (for droplet generation, Fig. S6a in Additional file 1) or two inlets (for the double emulsion, Fig. S6b). The designs for these chips are freely available to download as CAD-compatible or png files from the DropBase Repository of droplet microfluidic device designs [99]. The masters were built by applying a layer of SU-2025 (15 or 20 μm) on a 3 silicon wafer using conventional lithography. The master was coated with a mixture of poly(dimethyl)siloxane (PDMS, Slygard 184) and curing agent (Slygard 184) in a ratio of 10:1 (w/w). After degassing and curing (overnight, 65 °C), the PDMS devices were removed from the master, and holes for tubing connections were created using a biopsy punch (1 mm, PFM). The devices were then bonded onto a glass slide by treating with oxygen plasma (20s, Diener Femto plasma asher). For the hydrophobic chips, the triple inlet device was immediately filled with a 1% (v/v) Trichloro(1H,1H,2H,2H-perfluorooctyl)silane (FOTS) in HFE-7500 solution to provide a hydrophobic coating. The hydrophobic chips were incubated to ensure good bonding between PDMS and glass (65 °C, O/N). Once bonded, the double inlet devices were incubated to improve bonding (65 °C, 2 h), they were then treated with oxygen plasma a second time and immediately perfused with PBS to make the chips hydrophilic. The hydrophilic devices were kept in a hermetically sealed and humid box and were used within 1 week.

#### Metagenomic library preparation

Clones were arrayed individually from micro plates on solid plates to avoid growth bias, which could have occurred in liquid culture. After overnight growth at 37 °C, the cells were scraped, retrieved in LB medium supplemented with 12.5 μg/mL chloramphenicol and 8% (w/v) glycerol, and stored at -80 °C. In order to refresh the clones before screening, 250 μL were plated on LB agar medium supplemented with 12.5 μg/mL chloramphenicol, and grown overnight at 37 °C. All the colonies obtained were scraped off the plate and the plate was washed three times with 3 × 3 mL liquid LB containing 12.5 μg/mL chloramphenicol. The cells were harvested by spinning them down to form a pellet (4000 g for 10 min), the supernatant was discarded and the cells re-suspended in recovery medium (F98226-1, Lucigen). The OD_600nm_ of the cell suspension was measured and diluted in buffer (100 mM Tris-HCl pH 8 or 9, 100 mM NaCl, 12.5 μg/mL chloramphenicol and EDTA-free protease inhibitor, Roche) containing Percoll at 25% (v/v) in order to avoid aggregation of cells. This cell suspension was fed into the microfluidic device to form droplets (see details below) with a droplet occupancy (*λ*) of 0.35 when diluted 1:1 on chip with substrate (resorufin-β-GalNAc at 40 μM) in a 2 pL droplets following a Poisson distribution (resulting in about 70.5% of empty droplets, 25% with single cell, and 4.5% with higher occupancy).

#### Generation of single emulsions

For the generation of monodisperse microdroplets, a microfluidic device with a double aqueous inlet flow-focusing junction was used (Fig. S6a). The device was connected via polythene tubing (0.38 mm ID, 1.09 mm OD, Smiths Medical) to syringes (100 μL, 500 μL, or 2.5 mL SGE glass syringes), 2500 μL SGE which were driven by syringe infusion pumps (Nemesys low pressure pumps, Cetoni). Droplet formation was monitored using a high-speed camera (MiroEx2 Phantom camera, Vision Research). Water-in-oil single emulsions, containing resorufin-β-GalNAc (Fig. S6a inlet 2) and cells (Fig. S6a inlet 1), were formed in a hydrophobic flow-focusing device with channel dimensions of 15 μm height/120 μm width. The carrier oil phase was HFE 7500 (3 M) with 0.5% (w/w) surfactant (008-Fluorosurfactant, RAN biotechnologies) (Fig. S6a inlet 3). Flow rates were 1 μL/min total for the aqueous phases (cells and substrate) and 8 μL/min for the carrier oil phase. Immediately after the droplet generation, the droplets were stored in a 0.5 mL tube containing HFE-7500, 0.5% RAN surfactant (Fig. S6c and d).

#### Collection and incubation chambers

Collection chambers are a simpler version of a previously published design [100] (Fig. S6c and d). A 0.5-mL Eppendorf tube was inverted and glued onto a glass slide (Fig. S6c). Two holes were punctured using a 1 mm biopsy punch (PFM Medical), into which PTE tubing (0.38 mm ID × 1.09 mm OD) was inserted. The chamber is filled with carrier oil (HFE-7500, 0.5% RAN), and the tubing at the pinnacle of the tube connected to the chip outlet to collect the droplets (Fig. S6d1), with the lower tubing draining into an oil waste Eppendorf tube. Once the droplet generation was stopped, the lower tubing was inserted into a syringe which stops the flow. For re-injection (Fig. S6d2), the syringe was attached to a pump to allow the droplet to flow back up through the tubing and into a hydrophobic flow-focusing device (Fig. S6b).

#### Generation of double emulsions

Microfluidic double emulsions were generated as previously described [38] (Fig. S6e). The formation of water-in-oil-in-water double emulsions was performed in a hydrophilic microfluidic device (15 μm height/20 μm width, Fig. S6b). The aqueous carrier phase consisted of Tween 80 (1%, w/w) in 150 mM NaCl solution. The single emulsion was injection into the central inlet directly from the collection chamber. The double emulsion was collected in a 1.5-mL Eppendorf tube (Movie S1 in Additional file 3) and incubated at 37 °C for 24, 48, or 72 h, without agitation to allow cell growth.

#### Flow cytometric analysis of double emulsion droplets and cell recovery

Double emulsions were filtered (Ø 30 μm; sterile CellTrics®filters) into FACS tubes, diluted in buffer (Tween 80, 1%, w/w; 150 mM NaCl), and sorted by FACS (MoFlo Astrios, Beckman Coulter) using Tomato 561-579/16 for fluorescence selection. Flowing Software (version 2.5.0, Cell Imaging Core, Turku Centre for Biotechnology) was used for data analysis. Double emulsion populations were gated on logFSC/logSSC (Fig. S6f, gate R1). The sorted droplets (Fig. S6g, gate R3) were carefully de-emulsified by adding recovery medium (500 μL, containing 1% glycerol on top of the droplets) and breaking the emulsion by adding 50 μL of PFO to the oil phase. The recovered material was plated on LB agar plates containing 12.5 μg/mL chloramphenicol and incubated overnight at 37 °C.

### Protein production and purification

First, the Uhbg_GH123 encoding gene was PCR-amplified from the metagenomic clone 5 using the Phusion® high-fidelity DNA polymerase (New England Biolabs). The primers used were clone_5_ORF28_for (GGCAGCCATATGGCTAGCcaggcagaaaagtttccattgg) and clone_5_ORF28_rev (GTGGTGGTGGTGCTCGAGttacatctcattgagcaatgc). After PCR amplification, the open reading frame coding for the GH123 enzyme was subcloned into the pET28a expression vector using Gibson Assembly® Master mix. The recombinant plasmid pET28a_GH123 was used to transform *E. coli* BL21 star (DE3) competent cells. For protein expression, one individual colony was grown in LB (containing 50 μg/mL kanamycin) at 37 °C until the OD_600nm_ reached 0.5. Then, expression was induced by adding isopropyl-β-D-thiogalactoside (1 mM) for 4 h at 37 °C. Cells were harvested by centrifugation, re-suspended in purification buffer (20 mM TrisHCl pH 8, 300 mM NaCl) containing 0.5 mg.mL^−1^ lysozyme to reach an OD_600_nm of 80. After incubation at 37 °C for 1 h, the suspension was frozen 15 min at −80 °C and then defrosted. The samples were then centrifuged and the cell lysate was loaded on affinity chromatography resin (TALON®). Unbound proteins were washed with purification buffer containing 5 mM imidazole before final elution with buffer containing 200 mM imidazole. Protein concentration and buffer exchange was performed by using Millipore Amicon Ultra-15 Centrifugal Filter Concentrators (cut-off 10 kDa), and the elution buffer was changed to 50 mM sodium phosphate pH 7 for storage. The concentration of the purified protein was quantified using a NanoDrop™ 2000 spectrophotometer (Thermo Fisher Scientific) with an extinction coefficient ε of 151,635, determined using ProtParam [101].

### Enzymatic assays

#### Substrates

pNP-substrates were purchased from Carbosynth. GalNAcβ1-4Galβ1-4Glc (aGM2), GalNAcβ1-4(Neu5Acα2-3)Galβ1-4Glc (GM2), Galβ1-3GalNAcβ1-4(Neu5Acα2-3)Galβ1-4Glc (GM1a), GalNAcβ1-3Gal (tGb4), GlcNAcβ1-3Galβ1-4Glc (LNT2), Galβ1-3GlcNAcβ1-3Galβ1-4Glc (LNT), and Galβ1-4GlcNAcβ1-3Galβ1-4Glc (LNnT) were purchased from Elicityl. X-substrates (5-bromo-4-chloro-3-indolyl-β-GalNAc, and β-GlcNAc) were purchased from Carbosynth.

#### Functional profiling of the hit clones

After cell recovery on solid plates, the individual colonies were picked for growth in 96-deep well plates containing LB media with 12.5 μg/mL chloramphenicol for overnight incubation at 37 °C. First, the clones were grown in LB medium, supplemented with chloramphenicol 12.5 μg/mL, with orbital shaking (600 rpm for deep wells). Plate copy was performed using a Microlab NIMBUS workstation (Hamilton) and this step was repeated twice to homogenize the final OD value of the cultures. After 24 h, cells were harvested by centrifuging for 30 min at 5000 rpm., re-suspended in 300 μL 50 mM Na phosphate pH 7 buffer containing lysozyme (0.5 mg/mL final concentration) and DNase (0.3 U final concentration) and incubated at 37 °C for 1 h. The following steps were automated using the Genesis RSP 200 workstation (TECAN). Cell extracts were diluted tenfold in 50 mM Tris/HCl buffer (at pH 8 or 9) containing 20 μM resorufin-β-GalNAc, and incubated at 37 °C. The release of fluorescent product was monitored over 2 h using an Infinite M200 Pro (TECAN) plate-reader (excitation wavelength 550 nm and emission wavelength 585 nm). In addition, the hit clones were tested for activity on solid plates containing chromogenic glycosides of various structures. The hit clones stored in micro-plates were spotted on OmniTray™(Thermo Fisher Scientific) using a QPixII (Genetix) colony picker. Solid agar was supplemented with chloramphenicol (12.5 mg/mL) and 60 μg/mL of X-substrates (5-bromo-4-chloro-3-indolyl-β-GalNAc, 5-bromo-4-chloro-3-indolyl-β-GlcNAc). The plates were incubated 7 days at 37 °C. The positive clones were visually detected based on the blue color of the colony when X-substrates were degraded.

#### Quantification of Uhgb_GH123 activity on chromogenic substrates

First, the activity of the purified Uhgb_GH123 was quantified with several chromogenic substrates (pNP-β-D-N-acetylgalactosamine, pNP-β-D-N-acetylglucosamine, pNP-α-D-N-acetylgalactosamine, pNP-α-D-N-acetylglucosamine, pNP-α-D-glucopyranoside, pNP-β-D-glucopyranoside, pNP-α-D-galactopyranoside, pNP-α-D-mannopyranoside, and pNP-β-D-mannopyranoside), at 37 °C during 24 h in a reaction volume of 200 μL, by incubating 5 μL of Uhgb_GH123 (90 μM) in 50 mM sodium phosphate pH 7 buffer containing 1 mM of the pNP-substrate. Kinetic parameters of purified Uhgb_GH123 were determined by incubating in a 400 μL reaction mixture respectively 2 μL and 20 μL of purified Uhgb_GH123 (0.60 μM and 0.03 μM) with 0.25 to 1.5 mM of pNP-β-D-N-acetylgalactosamine and pNP-β-D-N-acetylglucosamine, in citrate buffer at pH 5.5, during 7 min and 60 min, respectively. The reaction was stopped by raising the pH to 11 by adding an equal volume of 0.2 M Na_2_CO_3_. The release of chromogenic product was monitored at 405 nm using an Infinite M200 Pro (TECAN) plate reader.

#### Human glycan degradation assays

The human glycan hydrolytic activities of the cell extracts of the metagenomic clones and of the purified Uhgb_GH123 were examined using HPAEC-PAD analysis. Enzymatic reactions were carried out at 37 °C in a final volume of 100 μL of 50 mM sodium phosphate pH 7 buffer, by mixing 50 μL of fosmid clones cell extracts (prepared as mentioned above) filtered at 0.22 μm, or 2 μM of purified Uhgb_GH123, with 2 mM glycoside. After 24 h incubation, the samples were heated 5 min at 95 °C to stop the reaction. Then the samples were diluted in water to reach a final substrate concentration of 200 mg/L for aGM2, LNT, LNnT, LNT2, and tGb4, and 400 mg/L for GM2 and GM1a, filtered at 0.22 μm and then analyzed using HPAEC-PAD on a Dionex ICS-3000 system (Dionex) equipped with a CarboPac PA100 column. The analyses were carried out at 30 °C with a flow rate of 1 mL/min and the following multi-step gradient: 0–15 min (0–60% B), 15–20 min (60% B), and 20–25 min (0% B). The solvents used were 150 mM NaOH (eluent A) and 150 mM NaOH, 500 mM CH_3_COONa (eluent B). GalNAc, GlcNAc, Gal, Glc, Neu5Ac, and lactose were used as standards.

### Sequencing

Clonal redundancy, defined as the ratio between the number of redundant clones and the total number of active clones, was determined by N- and C-terminal Sanger sequencing (GATC Biotech), after fosmid extraction from clones on agar plate supplemented with chloramphenicol (15 μg/mL). The primers used for N- and C-terminal sequencing were pCC1fos_forGGATGTGCTGCAAGGCGATTAAGTTGG and pCC1fos_revCTCGTATGTTGTGTGGAATTGTGAGC, respectively.

In order to perform full DNA insert sequencing, the fosmid DNA of the different clones identified after N- and C-terminal Sanger sequencing was extracted using the NucleoBondXtra Midi kit (Macherey-Nagel) according to the manufacturer’s instructions. Each purified DNA sample was assessed for quality by agarose gel electrophoresis giving one band only. The DNA concentration was measured using a NanoDrop™ 2000 spectrophotometer (Thermo Fisher Scientific). Sequencing of the fosmid DNA was performed by the GeT-Biopuces Platform (Toulouse) using the Ion Torrent S5 System. Read assembly was performed using Masurca [102]. The assembled contigs were cleaned from the pCC1FOS vector sequence using Crossmatch [103].

### Functional and taxonomical sequence annotation

The sequence similarities between clone hits were determined using NCBI-BLASTN [104]. The contig sequences were compared to sequences of the NCBI non-redundant (nr) database to determine the similarities with reference strain genomes. Taxonomic assignment of the metagenomic sequences was performed using the PhyloPythias program ([105], Model type Generic 2013–800 Genera). The open reading frames (ORFs) were predicted using the RAST annotation server [106]. Annotation of the CAZyme encoding genes was performed by CAZy curators following a procedure previously described [107]. First, BLASTP analysis of the predicted ORFs against the full-length sequences included in the CAZy database [107] allowed the automatic annotation of sequences that aligned over their entire length without gap, if > 50% identical. Second, the remaining sequences were manually analyzed by both (i) a BLAST search against individual GH, PL, CE, CBM, and GT modules and (ii) a HMMER3 search using Hidden Markov models built for each CAZy module family [108]. The most similar characterized glycoside-hydrolases (GHs) were identified by BLASTP comparison of the amino acid sequences of the GHs found in this study with those of the characterized members of their respective GH families, listed in the CAZy database. Signal peptide detection was performed with the LipoP server [109].

### Analysis of gene prevalence and abundance in the human fecal microbiome

The homolog sequences of the genes identified in this study were searched for in both translated catalogs of 9.9 and 22 million reference genes [51, 52] using BLASTP, *E* value = 0, identity ≥ 90%. The gene richness in the human gut was assessed by recovering the occurrence frequency data of homologous sequences of the catalog from the gene frequency table in the MetaHIT cohort [51]. This cohort includes a collection of 760 samples from European adults of various clinical status (healthy, and patients with Crohn’s disease or ulcerative colitis) and body mass index (lean, overweight, and obese) [110–112]. To examine if the gene prevalence was different between healthy versus IBD subjects, a Pearson’s chi-squared test was performed, based on the binary variable presence or absence of the gene in subjects.

## Supplementary information

**Additional file 1. **Supplemental information including additional figures and tables. **Fig. S1** Quantification of resorufin-β-GalNAc leakage between droplets at pH 8 and pH 9. **Fig. S2** Activity of the different clones isolated at pH 9, quantified in liquid medium on resorufin-β-GalNAc. **Fig. S3** SDS-PAGE analysis of cell extracts from clones 1 to 5 compared with control. **Fig. S4** HPAEC-PAD analysis of human glycans hydrolyzed by the different clones and by the purified Uhgb_GH123 enzyme. **Fig. S5** Abundance and prevalence in the human gut microbiome of European healthy and IBD affected individuals of the genes encoding the characterized GH123s. **Fig. S6** Illustrations for the microfluidics-based screening. **Table S1.** Summary of the clonal redundancy identified by Sanger sequencing of the metagenomic DNA extremities. **Table S2.** Activity of the different clones on X-β-GalNAc and X-β-GlcNAc. **Table S4.** Repetition of droplet-based microfluidic screening. **Table S5.** Summary of the kinetic parameters of the characterized members of the GH123 family, including the Uhgb_GH123. **Table S6.** Estimated costs of screening 90 x 20,000 clones or 1.8 .10^6^ experiments in microfluidic droplets compared to an automated robotic system.

**Additional file 2. Table S3.** Functional and taxonomical annotation of the metagenomic sequences.

**Additional file 3. Movie S1.** Generation of double emulsion droplets.

## Data Availability

The annotated contig sequences were deposited in the DDBJ/ENA/GenBank Nucleotide Sequence Database under accession numbers LR594826 (sequence of clone 1), LR594820 (clone 2), LR594811 (clone 3), LR594825 (clone 4), LR594812 (clone 5), LR594807 to LR594810 (clones 7 to 9), LR131278 (clone 10), LR594813 (clone 11), LR131285 (clone 12), LR594814 to LR594819 (clones 13 to 18), LR594821 to LR594824 (clones 19 to 22).

## Abbreviations

AADS: Absorbance-activated droplet sorting
AIEC: Adherent-invasive *E. coli*
BACs: Bacterial artificial chromosomes
β-GalNAcase: β-N-acetylgalactosaminidase
CAZymes: Carbohydrate active enzymes
CE: Carbohydrate esterases
IBD: Inflammatory bowel diseases
PL: Polysaccharide lyases
PULs: Polysaccharide utilization loci
PULDB: Polysaccharide utilization loci database
X-β-GalNAc: 5-bromo-4-chloro-3-indolyl-β-GalNAc
X-β-GlcNAc: 5-bromo-4-chloro-3-indolyl-β-GlcNAc
aGM2: GalNAcβ1-4Galβ1-4Glc
GM2: GalNAcβ1-4(Neu5Acα2-3)Galβ1-4Glc
GM1a: Galβ1-3GalNAcβ1-4(Neu5Acα2-3)Galβ1-4Glc
tGb4: GalNAcβ1-3Gal
LNT: Galβ1-3GlcNAcβ1-3Galβ1-4Glc
LNT2: GlcNAcβ1-3Galβ1-4Glc
LNnT: Galβ1-4GlcNAcβ1-3Galβ1-4Glc
