# Supplementary material for: Investigating host-microbiome interactions by droplet based microfluidics
Source: Microbiome. 2020 Oct 1;8:141. doi: 10.1186/s40168-020-00911-z (PMC7531118; doi:10.1186/s40168-020-00911-z)
Supplement: Supplementary file 2 — Additional file 1. Supplemental information including additional figures and tables. Fig. S1 Quantification of resorufin-β-GalNAc leakage between droplets at pH 8 and pH 9. Fig. S2 Activity of the different clones isolated at pH 9, quantified in liquid medium on resorufin-β-GalNAc. Fig. S3 SDS-PAGE analysis of cell extracts from clones 1 to 5 compared with control. Fig. S4 HPAEC-PAD analysis of human glycans hydrolyzed by the different clones and by the purified Uhgb_GH123 enzyme. Fig. S5 Abundance and prevalence in the human gut microbiome of European healthy and IBD affected individuals of the genes encoding the characterized GH123s. Fig. S6 Illustrations for the microfluidics-based screening. Table S1. Summary of the clonal redundancy identified by Sanger sequencing of the metagenomic DNA extremities. Table S2. Activity of the different clones on X-β-GalNAc and X-β-GlcNAc. Table S4. Repetition of droplet-based microfluidic screening. Table S5. Summary of the kinetic parameters of the characterized members of the GH123 family, including the Uhgb_GH123. Table S6. Estimated costs of screening 90 x 20,000 clones or 1.8 .106 experiments in microfluidic droplets compared to an automated robotic system. [file 40168_2020_911_MOESM1_ESM.doc]

**Supplementary information**

**Investigating host-microbiome interactions by droplet based microfluidics**

Alexandra S. Tauzin^1^, Mariana Rangel Pereira^2,3^, Liisa D. Van Vliet^2,4^, Pierre-Yves Colin^2^, Elisabeth Laville^1^, Jeremy Esque^1^, Sandrine Laguerre^1^, Bernard Henrissat^5,6,7^, Nicolas Terrapon^5,6^, Vincent Lombard^5,6^, Marion Leclerc^8^, Joël Doré^8,9^, Florian Hollfelder^2^,* and Gabrielle Potocki-Veronese^1^,*

^1^ TBI, CNRS, INRAE, INSAT, Université de Toulouse, F-31400 Toulouse, France.

^2^ Department of Biochemistry, University of Cambridge, CB2 1GA Cambridge, United Kingdom.

^3^ CAPES Foundation, Ministry of Education of Brazil, BrasÍlia – DF, Zip Code 70.040-020

^4^ Drop-Tech, Canterbury Court, CB4 3QU Cambridge, United Kingdom.

^5^ CNRS, UMR 7257, Aix-Marseille Université, F-13288 Marseille, France.

^6^ INRAE, USC 1408 AFMB, F-13288 Marseille, France.

^7^ Department of Biological Sciences, King Abdulaziz University, Jeddah, Saudi Arabia

^8^ Micalis Institute, INRAE, AgroParisTech, Université Paris-Saclay, F-78350 Jouy-en-Josas, France.

^9^ Metagenopolis, INRAE, F-78350 Jouy-en-Josas, France.

* Corresponding authors

Gabrielle Potocki-Veronese: [veronese@insa-toulouse.fr](mailto:veronese@insa-toulouse.fr)

Florian Hollfelder: fh111@cam.ac.uk

**Table of contents**

**Figure S1. Quantification of resorufin-β-GalNAc leakage between droplets at pH 8 and pH 9.**

**Figure S2. Activity of the non-redundant clones isolated at pH 9, quantified in liquid medium on resorufin-β-GalNAc.**

**Figure S3. SDS-PAGE analysis of cell extracts from clones 1 to 5 compared with control.**

**Figure S4. HPAEC-PAD analysis of human glycans hydrolyzed by the different clones and by the purified Uhgb_GH123 enzyme.**

**Figure S5. Abundance and prevalence in the human gut microbiome of European healthy and IBD affected individuals of the genes encoding the characterized GH123s.**

**Figure S6. Illustrations for the microfluidics-based screening.**

**Table S1. Summary of the clonal redundancy identified by Sanger sequencing of the metagenomic DNA extremities.**

**Table S2. Activity of the non-redundant clones on X-β-GalNAc and X-β-GlcNAc.**

**Table S4. Repetition of droplet-based microfluidic screening.**

**Table S5. Summary of the kinetic parameters of the characterized members of the GH123 family, including the Uhgb_GH123.**

**Table S6. Estimated costs (in USD) of screening 90 x 20,000 clones or 1.8.10^6^ experiments in microfluidic droplets compared to an automated robotic system.**

**
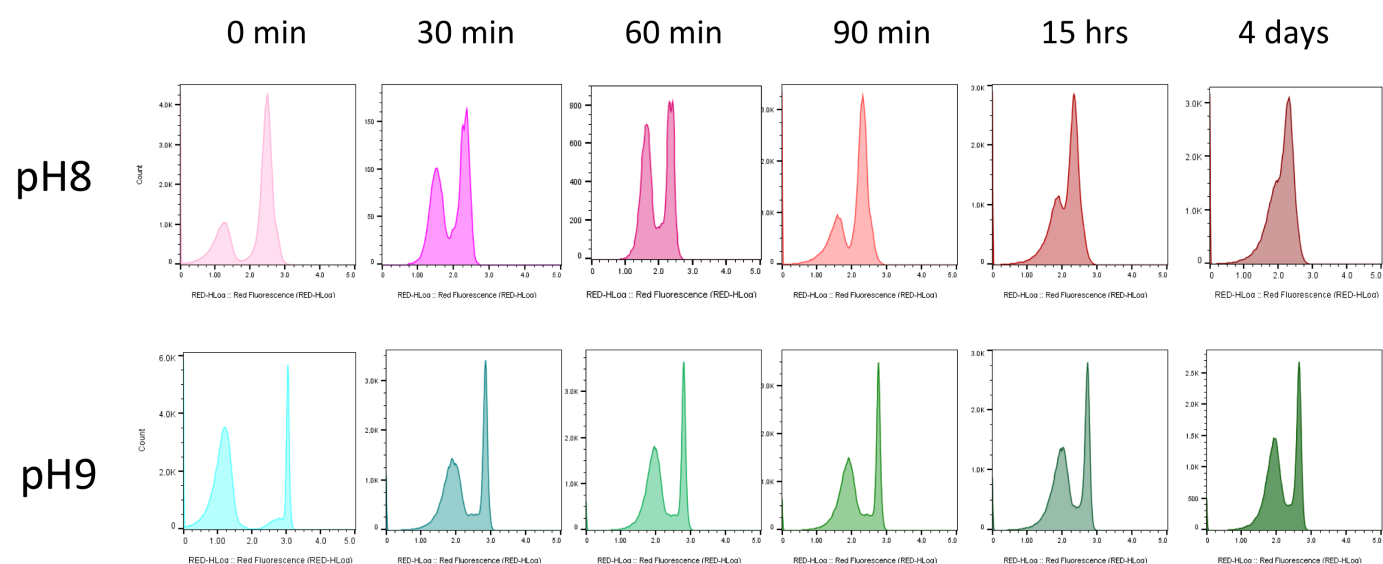
**

**Fig. S1 Quantification of resorufin-β-GalNAc leakage between droplets at pH 8 and pH 9.** FACS histograms for an equal volume of buffer droplets and droplets containing the substrate (resorufin-β-GalNAc), at either pH 8 or pH 9. The substrate emits fluorescence at 488 nm, with higher fluorescence at pH 9. The droplets were analysed by flow cytometry after incubation (37°C, for various times). These profiles show that resorufin-β-GalNAc leaks from the substrate droplets into buffer droplets at pH 8 from 30min, but that the two populations of droplets remain intact at pH 9 over at least 4 days.


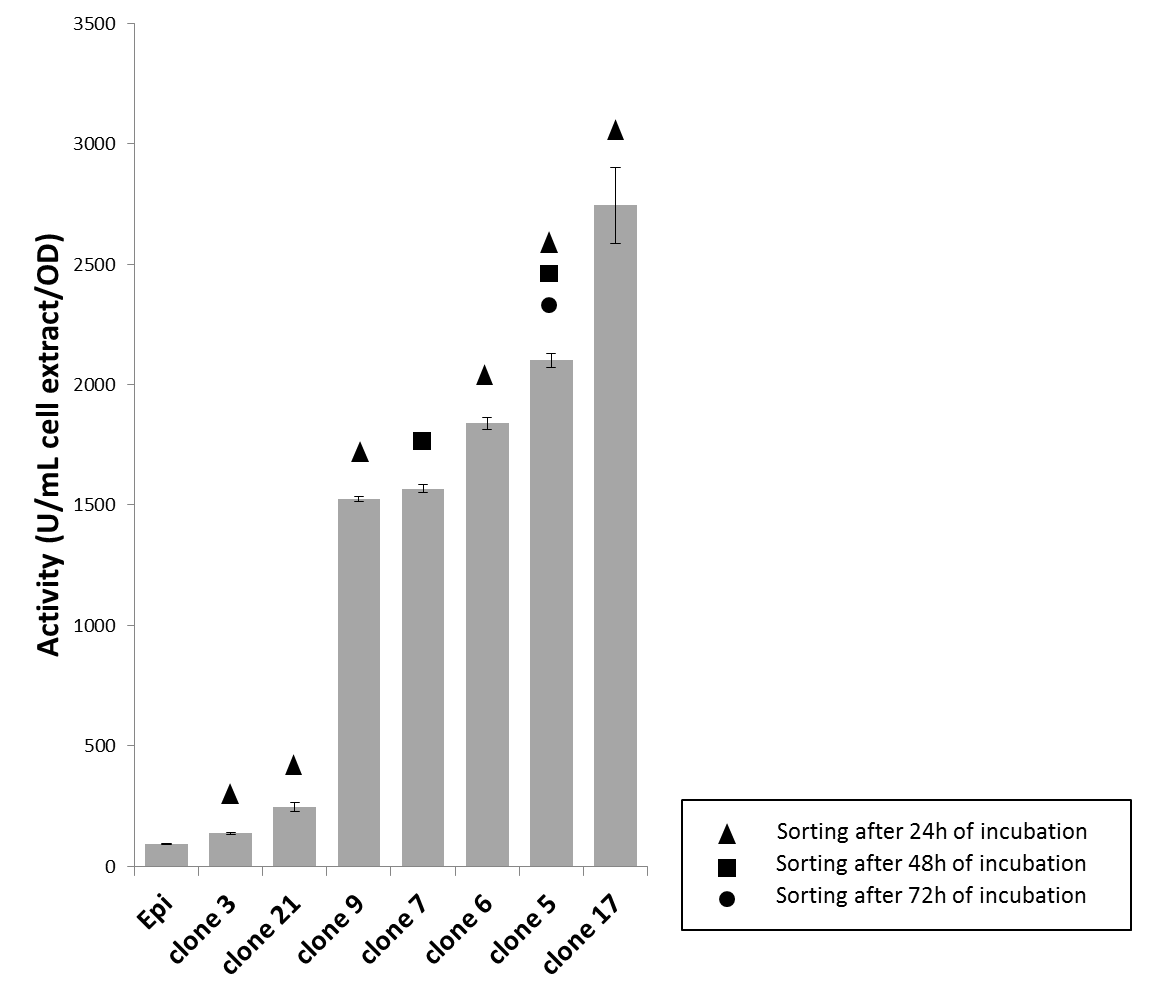


**Fig. S2** **Activity of the non-redundant clones isolated at pH 9, quantified in liquid medium on resorufin-β-GalNAc.** Activity was quantified at 37°C in 50 mM Tris/HCl, pH 9. The negative clone Epi (host Epi100 *E. coli* strain) was used as negative control.

***
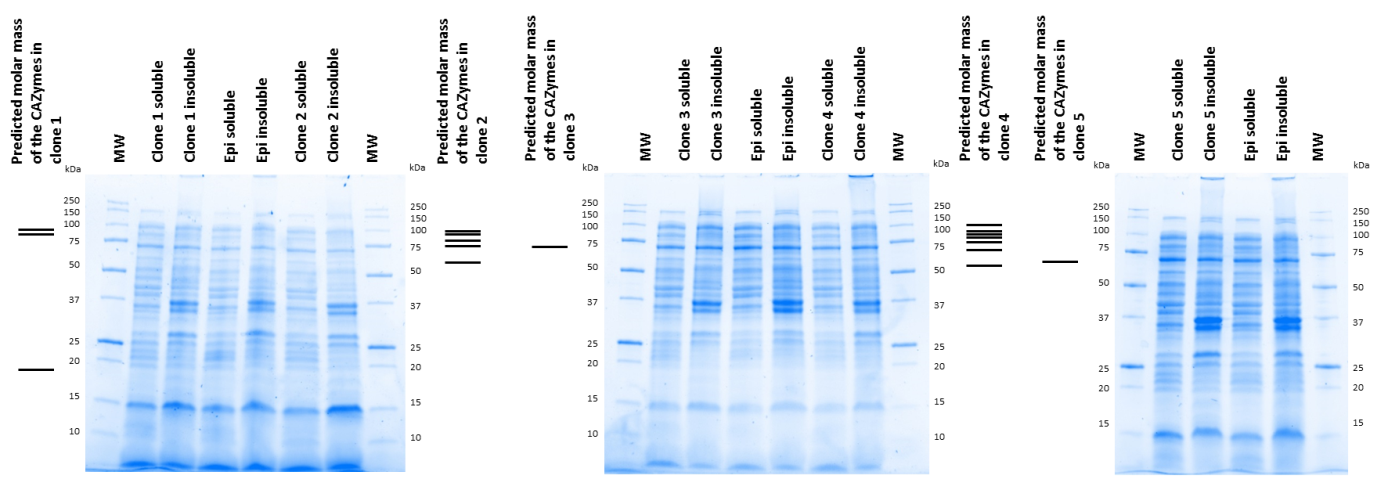
***

**Fig. S3 SDS-PAGE analysis of cell extracts from clones 1 to 5 compared with control.** Both the soluble and insoluble fractions were loaded onto the gels. The predicted molar masses of the CAZymes in the metagenomic clone are indicated on the side of the gels.

***
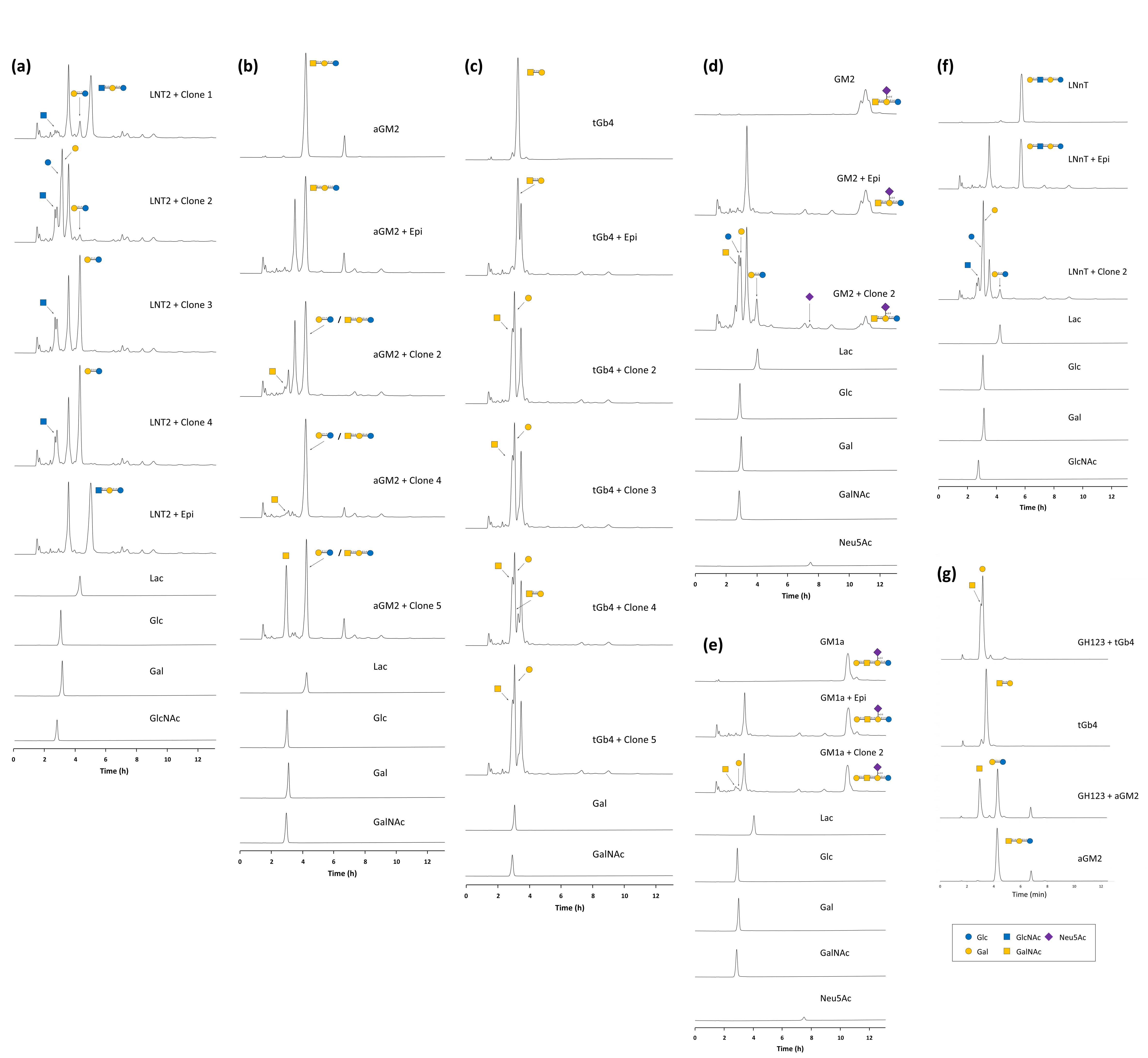
***

**Fig. S4** **HPAEC-PAD analysis of human glycans hydrolyzed by the different clones (a to f) and by the purified Uhgb_GH123 enzyme (g).** Only the chromatograms corresponding to positive reactions are presented. Cell extracts were incubated with 2 mM host-derived oligosaccharides for 24h at 37°C in 50 mM sodium phosphate buffer pH 7, before analysis by HPAEC-PAD (summarized in Table 2). The tested oligosaccharides, which share structural homologies with intestinal mucins, were the aGM2, GM2, tGB4 and GM1a ganglioside sugars, and the HMOs Lacto-N-triose (LNT2), Lacto-N-tetraose (LNT), Lacto-N-neotetraose (LNnT). The *E. coli* Epi100 screening strain constitutes the negative control. The purified Uhgb_GH123 enzyme isolated in clone 5 was tested under the same conditions on these substrates, using 2 µM of enzyme.


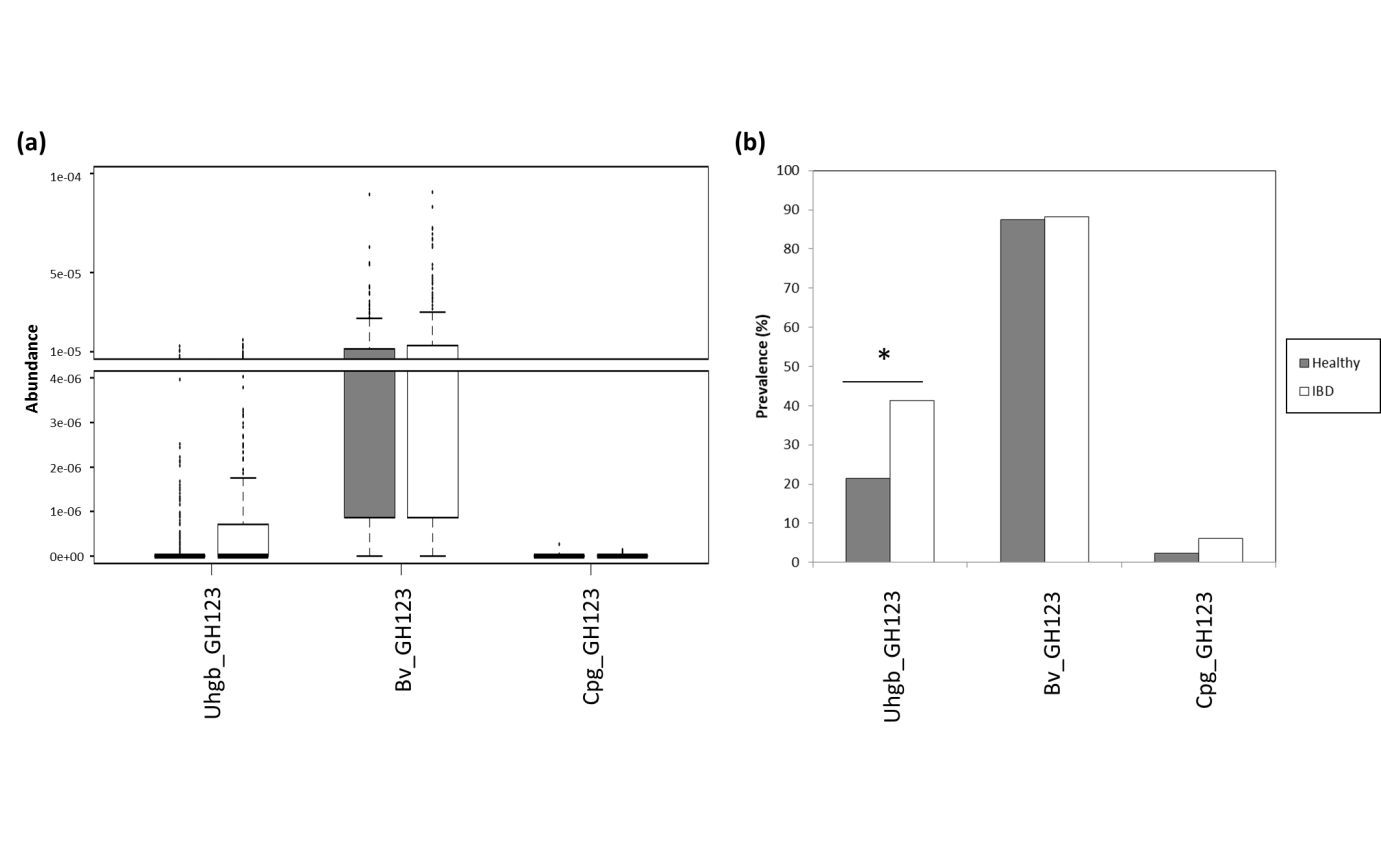


**Fig. S5 Abundance (a) and prevalence (b) in the human gut microbiome of European healthy and IBD affected individuals of the genes encoding the characterized GH123s.** Gene abundance and prevalence was determined in the gut metagenome of 760 European subjects including 401 healthy individuals and 359 IBD affected individuals. When the difference of prevalence between two clinical statuses is statistically significant, an asterisk is indicated beside the bars with a p-value < 10^-5^.

***
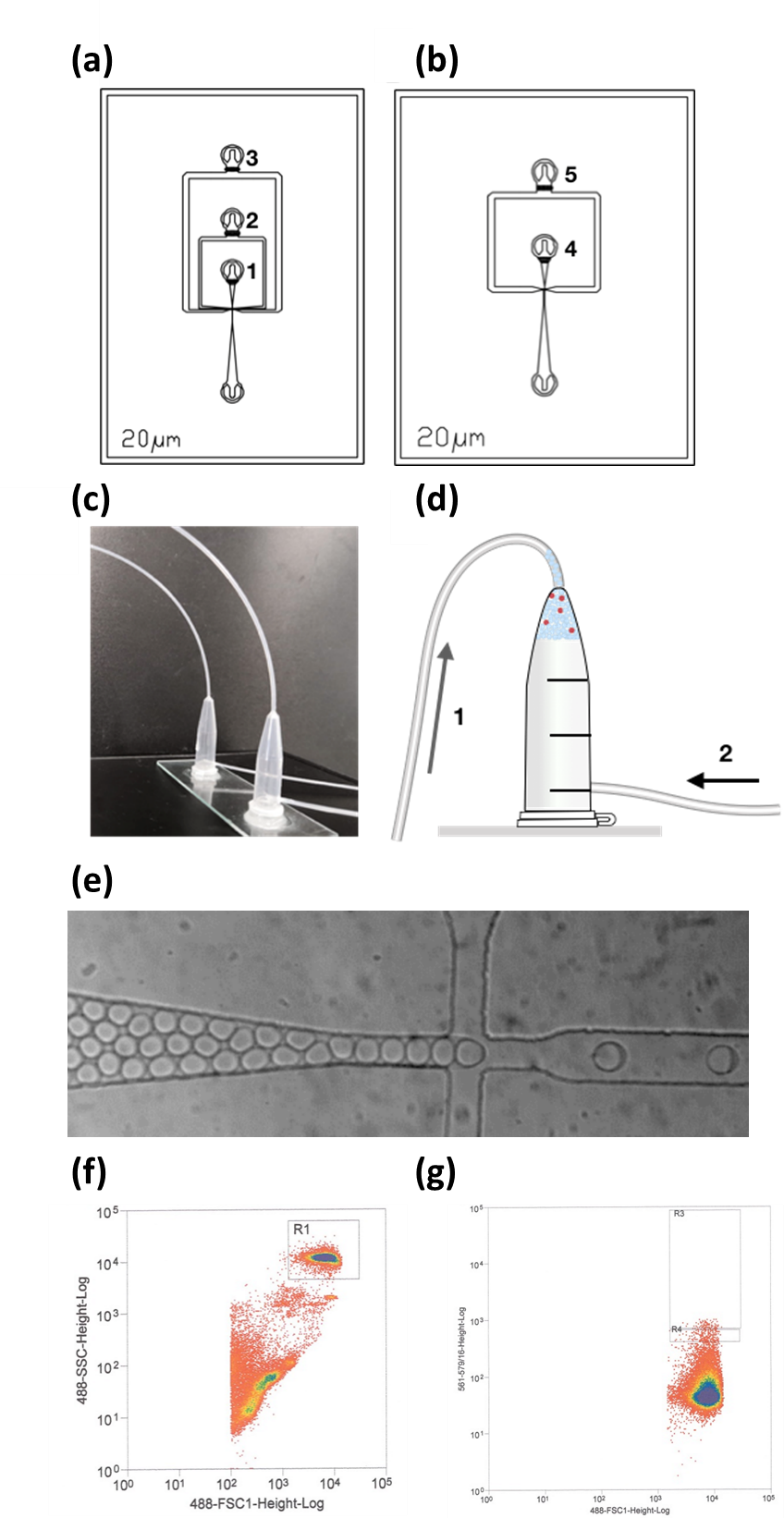
***

**Fig. S6 Illustrations for the microfluidics-based screening.** **Designs of the microfluidic chips (a and b).** Twenty µm intersection width, 15 µm channel height, used for generating (**a**) the single emulsion, with inlets for cells (1), for substrate (2) and for the carrier oil phase (3); and (**b**) double emulsion (water-oil-water) by re-injecting the droplet emulsions (4) into an aqueous phase (5). **Droplet collection and reinjection chamber (c and d).** (**c**) An image of two chambers. (**d**) A schematic of the droplet collection process, where (1) droplets are collected into oil and surfactant and (2) these can be pushed back out for reinjection into the hydrophobic chip for generation of the double emulsion. **Image of the double emulsion generation** (20x objective) (**e**). Water-in-oil droplets (in HFE-7500, 0.5% RAN surfactant) are injected into an aqueous flow (150 mM NaCL, 1% Tween80) to generate water-in-oil-in-water droplets. **FACS plots (f and g)** showing (**f**) FSC vs SSC with the double emulsion droplets in gate R1 and (**g**) the FSC vs Fluorescence (Tomato 561-579/16), where the top fractions of high fluorescence (R3 and R4) were collected for recovery.

**Table S1. Summary of the clonal redundancy identified by Sanger sequencing of the metagenomic DNA extremities.** All clones identified at pH 9 were also identified at pH 8. Under both pH conditions, the clone 5 was the most redundant, probably because of its high stability and high activity on the screening substrate both at pH 8 and 9. Clone 2, clone 8, clone 10 and clone 22 were inactive at pH 9, so instead they were sorted at pH 8. Clone 18 and clone 20 were isolated at the early time point (24h), but not after 48h or 72h incubation, probably because the ratio between their lysis and growth rates exceeded that of other clones cultivated under the same conditions. No correlation between time of incubation in the droplets and activity level was observed.

| **Clone name** | **Library screened at pH 8** | | | | | | | | | | **Library screened at pH 9** | | | | | | | |
| --- | --- | --- | --- | --- | --- | --- | --- | --- | --- | --- | --- | --- | --- | --- | --- | --- | --- | --- |
|  | **Repeat experiment** | | **Experiment** | | | | | | **Total** | | **Experiment** | | | | | | **Total** | |
|  | **Active clones 24h** | **Redundancy^b^ (%)** | **Active clones 24h** | **Redundancy^b^ (%)** | **Active clones 48h** | **Redundancy^b^ (%)** | **Active clones 72h** | **Redundancy^b^ (%)** | **Active clones Total** | **Redundancy^a^ (%)** | **Active clones 24h** | **Redundancy^b^ (%)** | **Active clones 48h** | **Redundancy^b^ (%)** | **Active clone 72h** | **Redundancy^b^ (%)** | **Active clones Total** | **Redundancy^c^ (%)** |
| Clone 1 |  |  |  |  |  |  | 1 | 4.8 | 1 | 1.0 |  |  |  |  |  |  |  |  |
| Clone 2 |  |  |  |  | 2 | 6.5 |  |  | 2 | 2.0 |  |  |  |  |  |  |  |  |
| Clone 3 | 3 | 7.9 | 2 | 4.0 | 2 | 6.5 | 3 | 14.3 | 7 | 6.9 | 1 | 2.9 |  |  |  |  | 1 | 2.4 |
| Clone 4 |  |  |  |  | 1 | 3.2 |  |  | 1 | 1.0 |  |  |  |  |  |  |  |  |
| Clone 5 | 11 | 28.9 | 13 | 26.0 | 4 | 12.9 | 4 | 19.0 | 21 | 20.6 | 16 | 47.1 | 4 | 80 | 3 | 100 | 23 | 54.8 |
| Clone 6 | 9 | 23.7 | 7 | 14.0 | 1 | 3.2 | 2 | 9.5 | 10 | 9.8 | 10 | 29.4 |  |  |  |  | 10 | 23.8 |
| Clone 7 | 1 | 2.6 | 2 | 4.0 | 1 | 3.2 | 1 | 4.8 | 4 | 3.9 |  |  | 1 | 20 |  |  | 1 | 2.4 |
| Clone 8 | 1 | 2.6 | 1 | 2.0 | 2 | 6.5 |  |  | 3 | 2.9 |  |  |  |  |  |  |  |  |
| Clone 9 | 3 | 7.9 | 5 | 10.0 | 1 | 3.2 |  |  | 6 | 5.9 | 5 | 14.7 |  |  |  |  | 5 | 11.9 |
| Clone 10 | 2 | 5.3 | 1 | 2.0 |  |  | 2 | 9.5 | 3 | 2.9 |  |  |  |  |  |  |  |  |
| Clone 11 |  |  |  |  |  |  | 1 | 4.8 | 1 | 1.0 |  |  |  |  |  |  |  |  |
| Clone 12 |  |  |  |  | 3 | 9.7 |  |  | 3 | 2.9 |  |  |  |  |  |  |  |  |
| Clone 13 | 1 | 2.6 |  |  | 1 | 3.2 |  |  | 1 | 1.0 |  |  |  |  |  |  |  |  |
| Clone 14 | 2 | 5.3 | 4 | 8.0 | 1 | 3.2 |  |  | 5 | 4.9 |  |  |  |  |  |  |  |  |
| Clone 15 | 2 | 5.3 | 3 | 6.0 | 5 | 16.1 |  |  | 8 | 7.8 |  |  |  |  |  |  |  |  |
| Clone 16 |  |  | 2 | 4.0 | 4 | 12.9 |  |  | 6 | 5.9 |  |  |  |  |  |  |  |  |
| Clone 17 |  |  |  |  |  |  | 2 | 9.5 | 2 | 2.0 | 1 | 2.9 |  |  |  |  | 1 | 2.4 |
| Clone 18 |  |  | 3 | 6.0 |  |  |  |  | 3 | 2.9 |  |  |  |  |  |  |  |  |
| Clone 19 |  |  | 3 | 6.0 |  |  | 5 | 23.8 | 8 | 7.8 |  |  |  |  |  |  |  |  |
| Clone 20 |  |  | 2 | 4.0 |  |  |  |  | 2 | 2.0 |  |  |  |  |  |  |  |  |
| Clone 21 |  |  | 2 | 4.0 | 2 | 6.5 |  |  | 4 | 3.9 | 1 | 2.9 |  |  |  |  | 1 | 2.4 |
| Clone 22 | 1 | 2.6 |  |  | 1 | 3.2 |  |  | 1 | 1.0 |  |  |  |  |  |  |  |  |
| Clone 23* | 2 | 5.3 |  |  |  |  |  |  |  |  |  |  |  |  |  |  |  |  |
| **Total** | 38 | 100 | 50 | 100 | 31 | 100 | 21 | 100 | 102 | 100 | 34 | 100 | 5 | 100 | 3 | 100 | 42 | 100 |

*^a^Overall redundancy (%) =*
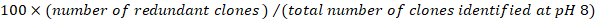


*^b^Redundancy (%) =*
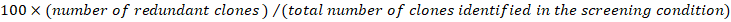


*^c^Overall redundancy (%) =*
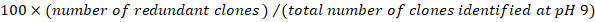


** Clone identified during the repeat experiment performed at pH 8, sharing synteny with the clone 3 and including a GH20 identical to the clone 3_GH20.*

**Table S2. Activity of the different clones on X-β-GalNAc and X-β-GlcNAc. The different clones were plated on LB agar supplemented with chloramphenicol (12.5 mg/mL) and 60 µg/mL of X-substrates (5-bromo-4-chloro-3-indolyl-β-GalNAc or 5-bromo-4-chloro-3-indolyl-β-GlcNAc). Then the plates were incubated 7 days at 37°C. The positive clones were visually detected based on the blue color of the colony when X-substrates were degraded.**

| **Clone name** | **Clone 1** | **Clone 2** | **Clone 3** | **Clone 4** | **Clone 5** | **Clone 6** | **Clone 7** | **Clone 8** | **Clone 9** | **Clone 10** | **Clone 11** | **Clone 12** | **Clone 13** | **Clone 14** | **Clone 15** | **Clone 16** | **Clone 17** | **Clone 18** | **Clone 19** | **Clone 20** | **Clone 21** | **Clone 22** | **Control** |
| --- | --- | --- | --- | --- | --- | --- | --- | --- | --- | --- | --- | --- | --- | --- | --- | --- | --- | --- | --- | --- | --- | --- | --- |
| X-β-GalNAc | **-** | **-** | **-** | **X** | **-** | **-** | **-** | **-** | **-** | **-** | **-** | **X** | **X** | **-** | **X** | **X** | **-** | **X** | **-** | **-** | **X** | **-** | **-** |
| X-β-GlcNAc | **X** | **X** | **-** | **X** | **-** | **-** | **-** | **X** | **-** | **X** | **-** | **X** | **X** | **-** | **X** | **X** | **-** | **X** | **-** | **-** | **X** | **-** | **-** |

**Table S4. Repetition of droplet-based microfluidic screening.** After 24h of incubation at pH 8, FACS sorting of the positive droplets and de-emulsification, cells were plated on solid agar to recover the sorted clones. For each screening experiment, the activity towards resorufin-β-GalNAc of 372 clones recovered on solid plates was quantified at 37°C in 50 mM Tris/HCl at pH 8 in order to select those with at least twice the activity of the *E. coli* host used as negative control. Then, the clonal redundancy resulting from the bulk de-emulsification of the hit droplets was quantified by sequencing the metagenomic insert extremities for each positive clone. From these sequences, the yields of different positive clones isolated from the initial metagenomic library of 20,000 clones were determined.

|  | **Number of sorted droplets by FACS** | **Number of colonies on agar plate** | **Number of tested clones** | **Sequenced clones** | **Different positive clones** | **Positive clone yield^a^ (‰)** |
| --- | --- | --- | --- | --- | --- | --- |
| **Experiment 1** | | | | | | |
| **24h** | 2.7 k | ND | 372 | 38 | 12 | 0.6 |
| **Experiment 2** | | | | | | |
| **24h** | 5 k | 7312 | 372 | 50 | 14 | 0.7 |

*^a^Positive clone yield (‰) =*
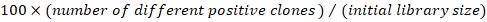


*ND: not determined.*

**Table S5. Summary of the kinetic parameters of the characterized members of the GH123 family, including the Uhgb_GH123.** Uhgb_GH123 reactions were performed at its optimal pH (5.5) and temperature (30°C), in the presence of 0.25 to 1.5 mM of *p*NP-β-D-N-acetylgalactosamine and *p*NP-β-D-N-acetylglucosamine.

| **Protein name** | ***K*_m_ (mM)** | ***k*_cat_ (s^-1^)** | ***k*_cat_/*K*_m_ (M^-1^s^-1^)** | **Reference** |
| --- | --- | --- | --- | --- |
| NgaP *Paenibacillus sp.* | | | | |
| pNP-β-GalNAc | 0.35 | 7.3 | 20,857 | [46] |
| *Cp*Nga123 *Clostridium perfringens* | | | | |
| pNP-β-GalNAc | 0.261 ± 0.043 | 181 ± 11 | 693,487 | [47] |
| *Bv*GH123 *Bacteroides vulgatus* | | | | |
| pNP-β-GalNAc |  |  | 8,000 | [45] |
| pNP-β-GlcNAc |  |  | 380 |  |
| Uhgb_GH123 *Uncultured human gut bacterium* | | | | |
| pNP-β-GalNAc | 1.17 ± 0.10 | 21.25 ± 1.18 | 18,162 | Present work |
| pNP-β-GlcNAc | 3.87 ± 0.27 | 0.33 ± 0.02 | 85 |  |

**Table S6. Estimated costs (in USD) of screening 90 x 20,000 clones or 1.8.10^6^** experiments in microfluidic droplets compared to an automated robotic system.

|  | **Microfluidic droplets** | | **Robotic system*** | |
| --- | --- | --- | --- | --- |
| **Initial investment in instrumentation** | **$39,000 **** | | **$300-750,000** | |
| **Total consumables costs** | **$ 349** | | **$ 127,747** | |
|  | **Quantities** | **Price (USD)** | **Quantities** | **Price (USD)** |
| **HFE 7500** | **5mL** | **$1.5** | **-** | **-** |
| **Surfactant** | **40 mg** | **$33** | **-** | **-** |
| **Tubing** | **3.2 m** | **$5** | **-** | **-** |
| **Chip master mould***** | **2 designs** | **$100** |  |  |
| **PDMS chip** | **2 chips** | **$0.6** | **-** | **-** |
| **Chip coatings** | **1 hydrophilic,**  **1 hydrophobic** | **$4.4** | **-** | **-** |
| **SOC** | **500µL** | **$0.5** | **-** | **-** |
| **Substrate for assay** | **8.3 µg** | **$0.09** | **1.5 g** | **$16,029** |
| **LB for profiling 372 hit clones** | **37 mL** | **$1.6** | **180L** | **$7,560** |
| **Substrate for profiling 372 hit clones** | **309 µg** | **$3.3** | **-** | **-** |
| **Multi-well plates for validation screening** | **8 x 96-well plates** | **$11.6** | **4,688 x 384-well plates**  **18,750 x 96-well plates** | **$50,158** |
| **Tips (1-200µL)** | **1,146** | **$12** | **5,400,000** | **$54,000** |
| **FACS facility use** | **2 hrs** | **$175** | **-** | **-** |

** Estimates with 2020 prices and GBP/USD exchange rate at time of final submission. Similar to [30].*

*** Not including a flow cytometer which may be available as a shared facility in many institutions.*

*** The authors have made their own master moulds but these are also commercially available for ~ $400.*

**Comparison Robotics vs Microfluidics**

***Number of plates:***  *At least 18,750 sterile 96-well plates and 4,688 basic 384-well plates would be required for (i) growth and lysis and (ii) activity assay. Sterile 96-well plates for hit colony growth ($1.8/plate) cost $33,750. Basic non-sterile 384-well plates (polystyrene, $3.50 per plate) would be at least $16,408 for a robotic screening campaign. This gives a total of $50,158. For the profiling of hit clones following the sorting of microfluidic droplets, eight 96-well plates were used to grow and then characterize the 372 (per condition) picked clones. Sterile 96-well plates for hit colony growth cost $7.2. 96-well plate (non-sterile, polystyrene, $1.1/plate) cost $4.4 for 4 plates. This gives a total of $11.6 in multi-well plates.*

***Number of tips:*** *Assuming that for each clone, the procedure would use a minimum of three pipette tips for (i) culture seeding, (ii) addition of lysis buffer, (iii) transferring cell lysate into the assay plate, and that twice 384 tips would be used to fill the plates with growth media and substrate, this equates to 5.4 million tips at ~ $0.01/ tip (average basic tip price) or $54,000.*

*For the microfluidic screening: 14 tips were used to prepare the reagents and cells for the microfluidic assay: 3 tips for the washing steps, 3 to remove supernatant, 1 to measure the OD, 1 to dilute in buffer, 1 for addition of Percoll, 2 to dilute fluorinated oil and fluorosurfactant, 3 to make aqueous phase. For hit characterization: (i) 8 tips on a multi-channel for growth media, (ii) 372 tips for culture seeding (iii) 372 for adding the lysis buffer (iv) 8 tips to dispense substrate into the assay plates and (v) 372 tips for cell lysate transfer to the assay plate; a total of 1132. For the entire microfluidic screening and characterization 1146 tips were used, amounting to a cost of ~ $12 for tips.*

***Substrate:*** *Resorufin N-acetyl-β-D-galactosamine was purchased for $10.7/ mg with a MW of 416.38 g/mol.*

*Assuming a working assay volume in a 384-well of 100µL, screening 1.8 million clones would use 180 L of substrate at 20 µM (20 µmols/L x 180L= 3,600 µmols x 416.38 g/mol = 1,498 mg x $10.7/mg) would cost $16,029. For the microfluidic screening: 500 µL at 40 µM were prepared to screen 1.8 million bacteria cost $0.008 (500µL x 40µmol/10^6^ µL= 0.02 µmols x 416.38 g/mol= 8.32 µg = 0.0083 mg x $10.7/mg= $0.09). For the hit characterization: 100 µL of 20 µM substrate is equivalent to 0.83 µg/ $0.01 used for each of the 372 clones or $3.3 (309µg) in total.*

***Cost of microfluidic consumables:*** *The total cost in consumables, including, fluorous oil and surfactant ($34.5), tubing ($5), microfluidic chip fabrication and chip coating ($105), Recovery medium and LB ($8.5) comes to $182. The equivalent costs in media and plasticware for a robotic screen would come to over $111k.*
